# A Targeted Integration-Based CHO Cell Platform for Simultaneous Antibody Display and Secretion

**DOI:** 10.3390/antib14020038

**Published:** 2025-04-28

**Authors:** Jessica P. Z. Ng, Mariati Mariati, Jiawu Bi, Matthew Wook Chang, Yuansheng Yang

**Affiliations:** 1 Bioprocessing Technology Institute (BTI), Agency for Science, Technology and Research (A*STAR), 20 Biopolis Way, #03-01 Centros, Singapore 138668, Singapore; 2 Institute of Molecular Cell Biology (IMCB), Agency for Science, Technology and Research (A*STAR), 61 Biopolis Dr, #07-01 Proteos, Singapore 138673, Singapore; bi_jiawu@imcb.a-star.edu.sg; 3 Synthetic Biology Translation Research Programme, Yong Loo Lin School of Medicine, National University of Singapore, Singapore 117465, Singapore; 4 NUS Synthetic Biology for Clinical and Technological Innovation (SynCTI), National University of Singapore, 28 Medical Dr, #02-07 Centre for Life Sciences, Singapore 117456, Singapore

**Keywords:** CHO cells, antibodies, recombinase-mediated cassette exchange, manufacturability, functionality, simultaneous display and secretion

## Abstract

Objective: We developed a targeted integration-based CHO cell platform for simultaneous antibody display and secretion, enabling a streamlined transition from antibody library screening to production without requiring the re-cloning of antibody genes. Methods: The platform consists of a CHO master cell line with a single-copy landing pad, a helper vector expressing FLPe recombinase, and bi-functional targeting vectors. Recombinase-mediated cassette exchange was utilized to integrate targeting vectors into the landing pad. Bi-functional vectors were designed by incorporating a minimal furin cleavage sequence (mFCS), RRKR, and various 2A peptides between the heavy chain (HC) and a membrane anchor. Results: Incomplete cleavage at the mFCS and 2A sites facilitated the expression of both membrane-bound and secreted antibodies, while mutations in the 2A peptide produced a range of display-to-secretion ratios. However, a fraction of secreted antibodies retained 2A residues attached to the HC polypeptides. Further analysis demonstrated that modifying the first five amino acids of the 2A peptide significantly influenced furin cleavage efficiency, resulting in different display-to-secretion ratios for targeting vectors containing mFCS-2A variant combinations. To overcome this, we designed nine-amino-acid FCS variants that, when placed between the HC and membrane anchor, provided a range of display-to-secretion ratios and eliminated the issue of attached 2A residues in the secreted antibodies. Vectors with lower display levels proved more effective at distinguishing cells expressing high-affinity antibodies with closely matched binding affinities. The platform also demonstrated high sensitivity in isolating high-affinity antibody-expressing cells and supported robust antibody production. Conclusion: This targeted integration-based CHO platform enables efficient, in-format screening and production of antibodies with tunable display-to-secretion profiles. It provides a powerful and scalable tool for accelerating the development of functional, manufacturable therapeutic antibodies.

## 1. Introduction

Therapeutic immunoglobulin G (IgG) monoclonal antibodies have become the bestselling biopharmaceutical products on the market [1]. However, their development remains a technically challenging, time-consuming, and expensive process. The general antibody development workflow begins with the discovery phase, followed by production in mammalian cells and subsequent preclinical and clinical studies before market approval. Traditionally, antibody discovery has relied on either in vivo immunization of animals or in vitro display-based technologies. In vivo immunization triggers an immune response in animals, followed by the use of hybridoma or single B cell technologies to discover high-affinity, highly specific monoclonal antibodies [1,2]. On the other hand, in vitro display technologies, such as phage-, bacteria-, and yeast-display, identify high-affinity antibodies through high-throughput screening of immunized, naïve, and synthetic or semi-synthetic libraries [3]. Following this discovery, the identified antibodies are then cloned and expressed through transient transfections in mammalian cells to generate small quantities for affinity, specificity, and in vitro cell-based testing. Subsequently, lead antibodies are produced in large quantities using stably transfected Chinese hamster ovary (CHO) cells for further developability assessment, animal studies, and clinical trials. CHO cells have become the preferred choice for the mass production of therapeutic antibodies due to their high yield and ability to properly fold and assemble complex proteins with human-like glycosylations.

In the current process of antibody development, the transition from antibody discovery to production involves shifts in production strategies and host cells, such as transitioning from transient to stable production and changing from mouse cells and HEK293 cells to CHO cells. In addition, antibodies identified through microbial system-based display are usually in the form of single-chain variable fragment (scFv) or fragment antigen binding (Fab) fragments and lack natural post-translational modifications, necessitating conversion into full-length antibodies, the final format for production in mammalian cells. These modifications often result in changes to antibody function, expression level, and biophysical properties, which can increase both costs and timelines, along with the risk of failure [4,5]. Therefore, the development of novel technologies is needed to bridge the gap between antibody discovery and production.

The mammalian cell-based display technologies, which allow the screening of antibodies in their full-length format with authentic post-translational modifications, have become a more attractive alternative to microbial-based display systems [3,6,7,8,9,10]. Typically, these technologies display antibodies on the cell surface by genetically fusing the C-terminus of the heavy chain constant region to a membrane anchor domain, such as the human platelet-derived growth factor receptor (PDGFR) [11,12,13] or the glycophospholipid (GPI) transmembrane domains [14,15,16]. By double-staining the cell surface with antigens and antibodies against the constant regions of displayed antibodies, mammalian cell display enables flow cytometry-based screening of both binding affinity and expression levels of full-length antibodies [8]. Additionally, cell surface staining signals provide insights into biophysical properties such as aggregation propensity, polyreactivity, and thermostability [17,18,19]. Mammalian cell display captures all the expressed antibodies on the cell surface. However, screening for antibodies against difficult-to-express membrane proteins [20] and additional functionalities, such as cell activation and internalization, requires mammalian cell libraries that secrete antibodies [7,21,22,23,24,25]. Various microfluidic technologies—based on compartmentalization in droplets [24,26,27,28], nanostructures like nanopens [29], or microcapillaries [30]—have been developed to enable screening for these functionalities in combination with secretion-based mammalian cell libraries. In addition, large-scale antibody production for animal testing and developability assessments (e.g., stability, viscosity, and solubility) also requires secretion-mode expression.

To streamline the transition between display and secretion modes without re-cloning antibody genes, various technologies have been developed to enable the integration of antibody display and secretion within the same mammalian cell. These technologies can be grouped into two categories: (1) switchable display-to-secretion systems, which allow antibody expression to toggle between display-only and secretion-only modes, and (2) simultaneous display and secretion systems, which enable antibodies to be expressed concurrently both on the cell surface and in a secreted form. Switchable systems include approaches such as amber suppression [31], inducible fusion of protein A dual Z-domain with PDGFR transmembrane domain (ZZ-PDGFR) [7], chemically induced inhibition of furin cleavage [32], antibiotic-promoted translational readthrough of stop codons [33], the enzyme-cleavable surface-tethered all-purpose screening system (ECSTASY) [34], and “antibody-membrane switching” via recombinase-mediated DNA recombination [35,36,37]. The first four methods require the addition and removal of specific components—such as non-natural amino acids, gene expression inducers, furin enzyme inhibitors, or antibiotics—to toggle between display and secretion modes. This complexity can introduce workflow challenges and lead to cell-to-cell variability in display due to inconsistent induction. The latter two methods face limitations in achieving complete switching from display to secretion, due to low DNA recombination or enzyme cleavage efficiency. Additionally, switchable display-to-secretion systems lack flexibility in controlling display levels of antibodies, which may need to be adjusted depending on the application [38].

Simultaneous display and secretion systems include methods such as alternative splicing [22,23,39,40,41], leaky stop codons [42], minimal furin cleavage sequence (mFCS) [43], and 2A peptides [44]. Each approach comes with its own challenges. For instance, leaky stop codons may lead to low membrane-bound antibody display and a lack of control of display-to-secretion ratios. Similarly, adjusting the display-to-secretion ratio is challenging with the mFCS. Furin, a naturally occurring protease in the Golgi apparatus, sporadically cleaves the mFCS. Cleavage results in the secretion of antibodies, while the absence of cleavage leads to cell surface-displayed antibodies. The mFCS motif, composed of the conserved sequence R-X-K/R-R, offers limited engineering flexibility for tuning cleavage efficiency and thus the ratio of display-to-secretion [45,46]. Different strengths of splicing signals and 2A peptides have been developed to adjust the ratio of display to secretion [39,41,44]. However, cryptic splicing sites within the heavy chain (HC) gene may lead to the expression of incorrect products. Meanwhile, 2A peptides, which are 18–22 amino acids long, enable ribosomal skipping during translation to produce secreted antibodies. When skipping does not occur, antibodies remain surface-bound. Despite their utility, 2A peptides cleave at the glycine-proline bond, leaving residual fragments (16–20 amino acids) on secreted antibodies, potentially compromising the antibody’s biophysical and functional properties [47].

One more key consideration for developing technologies that streamline the transition from antibody discovery to production is the strategy for introducing antibody genes into mammalian cells. An ideal approach should meet several criteria: (1) high delivery efficiency to maximize library size, (2) expression of a single antibody gene per cell to allow efficient enrichment of clones with desired properties, (3) sustained expression to allow multiple rounds of enrichment and transition from antibody screening to production, and (4) high-level expression for generating enough material for testing complexed developability and functional properties. Transient expression, which has been historically used for the generation of mammalian cell libraries [48,49,50], fails to meet these requirements, as it provides short-term and low expression levels, and limited control over single-gene introduction per cell. Episomal plasmid vectors, which replicate without integrating into the chromosome, extend expression duration but still result in low expression levels and lack control over single-gene insertion per cell [15,39]. In contrast, stable integration offers both long-term and high-level expression. Methods for stable integration that have been used in the construction of mammalian cell libraries include random plasmid integration [15,35,38,43,51], viral-based transduction [12,20,36,52], transposons [22,23,53], and targeted integration. Random integration has low efficiency and poorly controls single-gene insertion per cell. Viral transduction and transposon-based methods enhance integration efficiency, yet they struggle with multiple-gene integration. In addition, these two approaches lack precise control over insertion sites, leading to transcriptional variation across different cells that complicates flow cytometry sorting. Targeted integration, achievable through recombinase- [13,18,19,37,54,55] or nuclease-mediated methods [56,57,58], has become the most favorable approach for constructing mammalian cell libraries. This strategy offers precise integration at desired loci, enabling transcriptional uniformity, effective single-gene insertion, and high expression levels, ultimately supporting efficient antibody screening and production.

Previous studies have utilized episomal plasmid vectors [39], transposon expression systems [22,23], viral transduction [44], and random integration [41,43] for achieving simultaneous display and secretion of monoclonal antibodies. However, the reported secreted titers from these technologies, ranging from 0.1 mg/L to 80 mg/L, are relatively low and insufficient to meet the requirements for testing antibody properties that demand larger quantities of antibodies. To address this limitation, we have previously developed a targeted integration-based CHO cell expression system that supports single-copy integration of targeting plasmid vectors into a predefined active genomic site through FLPe-aided recombinase-mediated cassette exchange (RMCE) [59]. This system enables rapid antibody production in stably transfected cell pools, reaching titers of several hundred mg/L in fed-batch cultures. In this study, we enhanced this system with a set of bi-functional vectors that enable simultaneous antibody display and secretion at different ratios. With the display function, this expression system facilitates high-throughput screening to efficiently identify high-affinity antibodies from a library. The secretion function allows CHO cells expressing high-binding antibodies to be further screened for functionality using microfluidic technologies. Subsequently, identified CHO cells expressing desirable antibodies can be directly used as production cell lines, generating sufficient material for further developability assessments and functional studies. By integrating both capabilities within the same CHO cells, this platform offers a powerful, streamlined solution for antibody discovery to production.

## 2. Materials and Methods

### 2.1. Construction of Targeting Vectors Expressing Single Antibodies and Antibody Libraries

**Figure 1:**
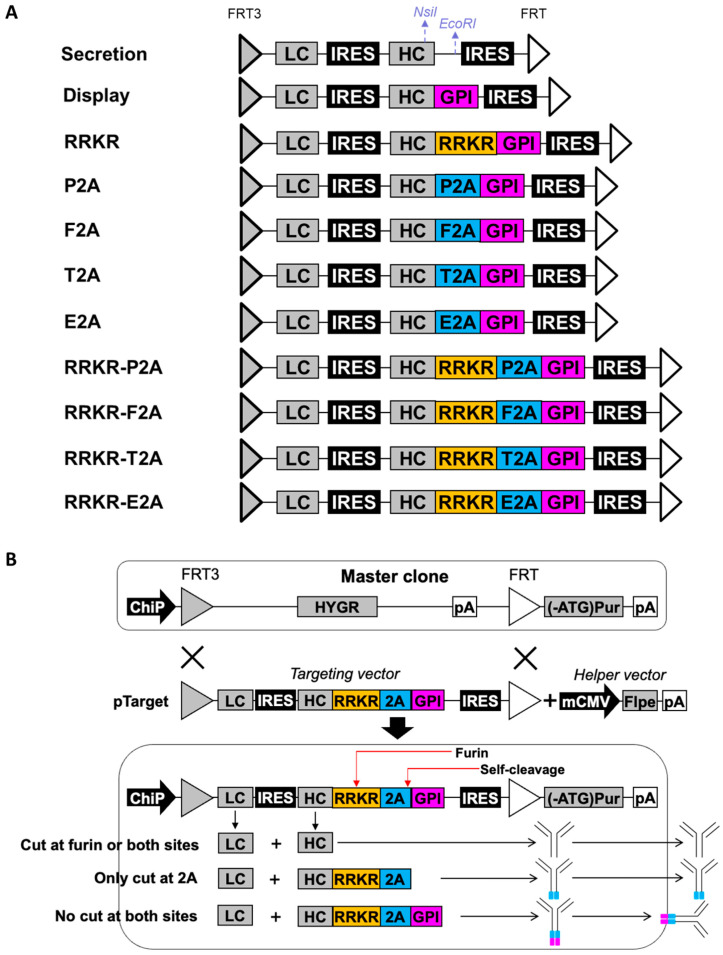
Targeting vectors, helper vectors, and recombinase-mediated cassette exchange (RMCE) for simultaneous antibody display and secretion. (**A**) Schematic representation of targeting vectors designed for antibody secretion only, display only, and simultaneous display and secretion. (**B**) Overview of RMCE-mediated integration of targeting vectors to enable simultaneous antibody display and secretion. Arrows indicate the initial translation, assembly, and transport of antibodies, either for secretion into the extracellular space or for display on the cell surface. The blue region represents the 2A peptide, while the pink region denotes the GPI membrane anchoring domain. ChiP, chimeric promoter comprising mCMV (cytomegalovirus) enhancer, human CMV core promoter and intron; FRT3 and FRT, mutated and wildtype flippase recognition targets; FLPe, enhanced recombinase flippase; HYGR, hygromycin resistance gene; pA, simian virus 40 polyadenylation signal; (-ATG) Pur, puromycin resistance gene lacking start codon ATG; IRES, wild-type encephalomyocarditis virus internal ribosomal entry site; mCMV, murine CMV enhancer and promoter; LC, light chain cDNA; HC, heavy chain cDNA; F2A, foot-and-mouth disease virus 2A peptide; E2A, equine rhinitis A virus 2A peptide; P2A, porcine teschovirus-1 2A peptide; T2A, Thosea asigna virus 2A peptide; GPI, glycosidylphosphatidylinositol membrane anchor from human decay-accelerating factor.

The secretion vector, containing the sequence FRT3-LC-IRES-HC-IRES-FRT, was synthesized by GenScript and served as the base vector for constructing other targeting vectors (Figure 1A). Additional targeting vectors in Figure 1A, Figure 3A and Figure 4A were created by inserting synthesized elements between the NsiI site at the 5′ end of the HC and the EcoRI site upstream of the second EMCV IRES in the base vector. Sequences for FLPe recombinase recognition sites (FRT3 and FRT), EMCV IRES, trastuzumab IgG1 LC and HC, P2A, F2A, E2A, T2A, and GPI have been previously described [14,15,16,47,59,60].

For the high- and low-display targeting vectors expressing antibodies of varying binding affinities, the high-affinity antibodies utilized the trastuzumab sequence (Figure 5A). Medium- and low-affinity humanized anti-HER2 antibodies were developed in-house. Their binding affinities were quantified using bio-layer interferometry assays on the Octet system (LakePharma). Targeting vectors expressing medium- and low-affinity antibodies were constructed by replacing the variable light chain (VL) and variable heavy chain (VH) regions in the trastuzumab-expressing high and low display vectors, using the SacII and BsiWI sites for VL and the NgoMIV and SalI sites for VH.

A humanized anti-HER2 antibody library was generated by shuffling the CDR of the murine anti-HER2 4D5 antibody with human germline framework sequences, as previously described [61]. The humanized VL and VH libraries were cloned into the low-display targeting vector containing the RRKRATNFP variant, using the SacII and BsiWI sites for the VL library and the NgoMIV and SalI sites for the VH library.

### 2.2. Generation and Characterization of Stably Transfected Cell Pools

The CHO K1 master cell line (MCL) was previously established by integrating a landing pad vector into CHO K1 cells (ATCC, Manassas, VA, USA) [59]. Protocols for culturing the MCL and generating stable cell pools via RMCE have been detailed in earlier work [59]. Stable cell pools were characterized for growth and productivity using 5-day batch or 12-day fed-batch cultures. Cultures were initiated at a seeding density of 3 × 10^5^ cells/mL in 30 mL protein-free medium consisting of HyQ-PF (GE Healthcare Life Sciences, Waltham, MA, USA) and CD-CHO (Thermo Fisher Scientific, Waltham, MA, USA) at 1:1 ratio, supplemented with 0.05% Pluronic F-68 (Thermo Fisher Scientific, Waltham, MA, USA), 1 g/L sodium bicarbonate (Sigma-Aldrich, St. Louis, MO, USA), and 6 mM L-glutamine (Sigma-Aldrich, St. Louis, MO, USA). For fed-batch cultures, 10% of Ex-cell Advanced CHO Feed 1 media (Sigma-Aldrich, St. Louis, MO, USA) was added every other day starting on day 3. Cell viability and density were assessed using a Vi-Cell XR viability analyzer (Beckman Coulter, Brea, CA, USA), and antibody secretion titers were measured with an IMMAGE 800 immunochemistry system (Beckman Coulter, Brea, CA, USA). Integrated viable cell density (IVCD) was calculated using the trapezoidal method. Specific antibody productivity (qMab) was determined by dividing the antibody concentration by the IVCD.

### 2.3. Cell Staining, Flow Cytometry Analysis, and FACS Enrichment

To evaluate targeting vectors with varying antibody display levels (Figure 1A), 1 × 10^7^ cells were collected, washed with 1×PBS, and incubated with 500 µL of a 1:100 dilution of Anti-Human IgG (γ-chain specific)–FITC (Sigma-Aldrich, St. Louis, MO, USA) on ice in the dark for 30 min. After staining, cells were washed twice with 1X PBS and resuspended at 5 × 10^6^ cells/mL for flow cytometric analysis using a FACSCalibur™ Flow Cytometer (BD Biosciences, Franklin Lakes, NJ, USA).

Both single-staining and double-staining methods were used to differentiate cells expressing antibodies with varying binding affinities. For single staining, 1 × 10^7^ cells were washed with 1× PBS and incubated with 50 µL of 10 µg/mL FITC-labeled Human Her2/ErbB2 Protein, His Tag (HER2-FITC) (ACROBiosystems, Newark, DE, USA) at room temperature in the dark for 30 min. For double staining, the same protocol was followed. Besides HER2-FITC, 50 µL of 100 µg/mL R-phycoerythrin (R-PE) AffiniPure F(ab’)_2_ Fragment Goat Anti-Human IgG, Fc Gamma-specific (HC-PE) (Jackson ImmunoResearch, West Grove, PA, USA) was also added during incubation. After staining, cells were either washed immediately before flow cytometry analysis (protocol with less dissociation) or resuspended in 1 mL of pre-warmed 1× PBS and incubated at 37 °C for 15 min to enhance antibody dissociation (protocol with enhanced dissociation). Cell washing was performed using 1 mL of 37 °C 1× PBS, followed by resuspension to a final concentration of 5 × 10^6^ cells/mL. Stained cells were analyzed using a FACSCalibur™ Flow Cytometer or sorted with a BD FACSAria™ III Cell Sorter (BD Biosciences). The sorting collection media comprised regular culture media supplemented with 1× Antibiotic-Antimycotic (Gibco, Waltham, MA, USA). Flow cytometry data were processed using FlowJo™ 10.7.2 (Tree Star Inc., Ashland, OR, USA).

### 2.4. Protein Purification, SDS-PAGE, and Peptide Mapping Analysis

Culture supernatants were purified using protein A on a GE AKTA Purifier 100 FPLC System (GE Healthcare, Chicago, IL, USA). The purified antibodies were analyzed under reducing conditions using SDS-PAGE, followed by peptide mapping of their amino acid sequences via NanoLC-MS/MS analysis. Protocols for these processes have been detailed in previous studies [47].

### 2.5. TOPO Cloning for HC and LC Sequences

RNA was extracted using the RNeasy Mini Kit (Qiagen, Hilden, Germany) following the manufacturer’s protocol. cDNA synthesis was performed with 2.5 µg of RNA using the EvoScript Universal cDNA Master (Roche, Basel, Switzerland). The LC and HC cDNA were independently amplified using the 2X Platinum SuperFi II Green PCR Master Mix (Thermo Fisher Scientific, Waltham, MA, USA) with primers listed in Supplementary Table S1. The purified PCR products were used for TA cloning with the TOPO™ TA Cloning™ Kit (Thermo Fisher Scientific, Waltham, MA, USA) for sequencing.

## 3. Results

### 3.1. Evaluation of mFCS, 2A Peptides, and Their Combinations for Simultaneous Display and Secretion of Full-Length IgG Antibody

Previous studies have utilized either mFCS or 2A peptides to achieve the simultaneous display and secretion of full-length IgG antibodies in mammalian cells [43,44]. However, engineering mFCS to adjust the display-to-secretion ratio presents significant challenges, while antibodies secreted using the 2A peptide approach retain residual 2A sequences, which may impact their properties. To achieve simultaneous antibody display and secretion while avoiding the secretion of products containing 2A residues, we designed a set of targeting vectors in which the LC gene is positioned as the first cistron and linked to the HC gene through an encephalomyocarditis virus (EMCV) internal ribosome entry site (IRES) element. The HC is further connected to a GPI membrane anchor via an mFCS (R-R-K-R) combined with one of four 2A peptides: foot-and-mouth disease virus 2A peptide (F2A), equine rhinitis A virus 2A peptide (E2A), porcine teschovirus-1 2A peptide (P2A), or Thosea asigna virus 2A peptide (T2A). The HC stop codon is removed, ensuring the HC, R-R-K-R, 2A, and GPI anchor are in a single reading frame. An additional EMCV IRES sequence is included downstream of the GPI sequence. The entire expression cassette is flanked by a pair of FLPe recombination targeting sites: wild-type FRT and mutated FRT3. These four vectors were named RRKR-P2A, RRKR-F2A, RRKR-T2A, and RRKR-E2A, respectively. For comparison, seven additional targeting vectors were designed with the same basic structure as those vectors described above. These variants differ in how the HC is linked to the GPI anchor: using only a 2A peptide, through R-R-K-R alone, directly fused to the GPI, or without the GPI anchor entirely. These seven vectors were named P2A, F2A, T2A, E2A, RRKR, display, and secretion, respectively (Figure 1A).

All targeting vectors were evaluated in stably transfected pools generated via RMCE using our previously developed CHO master clone. To establish stable pools, each targeting vector was co-transfected with a helper vector expressing FLPe into the CHO master cells, followed by selection in a medium containing puromycin. The CHO master clone contains a single-copy landing pad that expresses the hygromycin resistance (HYGR) gene flanked by FRT3 and FRT recombination sites matching those in the targeting vectors. Downstream of the FRT site, a start codon-deficient puromycin resistance gene ((ATG-)Pur) is located. The targeting vectors were designed without promoters and polyadenylation signals (pA). Accurate RMCE-mediated integration of the targeting vector into the landing pad activates the expression of the LC, HC (and its associated components), and the puromycin resistance gene, allowing the cells to survive selection. CHO master cells with incorrect integration fail to express these genes and are eliminated during selection. Simultaneous display and secretion functionality could be achieved in stable cell pools transfected with RRKR-2A vectors. These vectors enable antibody processing through furin cleavage at the RRKR site and/or ribosomal skipping at the 2A peptide site. This design results in the generation of three possible antibody forms: (1) secreted antibodies without attachment of 2A residues when cleavage occurs at the furin cleavage site or at both the furin cleavage site and the 2A peptide, (2) surface-displayed antibodies when neither furin cleavage nor ribosomal skipping of the 2A peptide occurs, and (3) secreted undesired antibodies when only ribosomal skipping occurs at the 2A peptide, resulting in antibodies with a redundant trailing amino acid sequence (Figure 1B).

**Figure 2:**
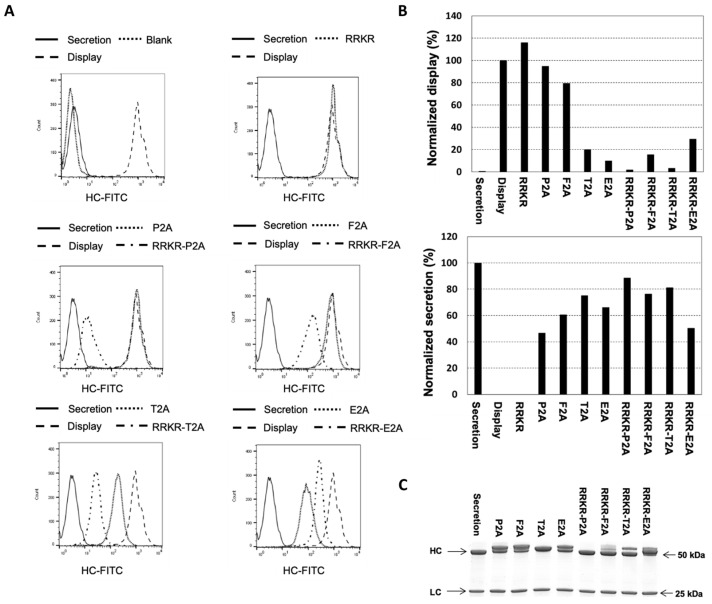
Evaluation of targeting vectors containing minimal furin cleavage sequence (RRKR), 2A peptides, and their combinations for antibody display and secretion. Stable cell pools were generated by co-transfecting CHO master cells with a specified targeting vector and a helper vector expressing FLPe recombinase, followed by selection in a puromycin-containing medium. Transfected cells were subsequently selected for correct integration of the targeting vectors into the landing pad. Non-transfected cells (blank), stable pools generated using the secretion vector, and those using the display vector served as controls. (**A**) Flow cytometry analysis of antibody levels displayed on the cell surface. Cells were stained with FITC-conjugated IgG specific to the heavy chain. (**B**) Normalized display and secretion levels relative to the control pools. Normalized display levels were calculated as the mean fluorescence intensity (MFI) of FITC-HC divided by that of the display control. Normalized secretion levels were determined as the qMab value from a 5-day batch culture relative to the secretion control. (**C**) SDS-PAGE analysis of purified secreted antibodies under reducing conditions.

Once stable pools were established, they were analyzed for surface antibody display using flow cytometry after staining with fluorescein isothiocyanate (FITC)-conjugated IgG specific to the heavy chain (FITC-HC) (Figure 2A). Antibody secretion levels were also evaluated for each stable pool in 5-day batch cultures. Two control pools were generated: one using the secretion vector (which lacks GPI and secretes all antibodies into the medium) and another using the display vector (which directly links the HC to GPI, resulting in all antibodies being displayed on the cell surface). The normalized display level was quantified as the mean fluorescence intensity (MFI) of pools generated using a specific targeting vector divided by the MFI from the display control. Similarly, the normalized secretion level was calculated as the specific productivity (qMab) of the pool generated using a specific targeting vector divided by the qMab from the secretion control (Figure 2B).

All targeting vectors exhibited homogeneous expression levels across cells, as indicated by sharp flow cytometry histograms. As expected, compared to non-transfected cells (blank), the secretion vector displayed negligible antibody levels on the cell surface, while the display vector demonstrated high levels of surface antibody display with no secretion. Interestingly, the RRKR vector achieved a display level slightly higher than the display control while exhibiting no secretion. This observation contrasts with a previous study that used the RIRR sequence to link the HC to a PDGFR membrane anchor, enabling simultaneous display and secretion of antibodies [43]. A potential explanation for this discrepancy lies in the differences in the membrane anchor and mFCS sequences used. Among the 2A vectors, P2A, F2A, T2A, and E2A displayed varying levels of surface antibody display, achieving 95%, 80%, 20%, and 10% of the display control, respectively. When combined with RRKR, all targeting vectors showed reduced display levels except for RRKR-E2A. The display levels observed from the RRKR-2A combinations did not directly correlate with those of their respective 2A vectors without RRKR. Specifically, RRKR-P2A exhibited the lowest display level, achieving only 2% of the display control. RRKR-T2A, RRKR-F2A, and RRKR-E2A exhibited higher display levels, achieving 4%, 18%, and 30% of the display control, respectively. A general inverse relationship between secretion and display levels was observed across the 2A and RRKR-2A-containing targeting vectors, with secretion levels ranging from 44% to 88% of the secretion control.

The secreted antibodies from different targeting vectors were purified using protein A and analyzed under reducing conditions on a denaturing SDS-PAGE gel (Figure 2C). As expected, the HC and LC polypeptides expressed from the secretion control vector were observed at approximately 50 kDa and 25 kDa, respectively. The LC polypeptides secreted from all other targeting vectors were also observed at approximately 25 kDa. However, for all four 2A peptide-containing targeting vectors, one or two bands with sizes larger than the HC polypeptide of the secretion control were observed. Peptide mapping analysis revealed that these HC species were attached with residual 2A or 2A-GPI peptide fragments (Supplementary Figure S1). The amount of HC-2A-GPI polypeptide present in the medium was small, which could have resulted from cell death. When RRKR was included upstream of F2A, T2A, and E2A, two distinct HC bands were still observed. However, the sizes of the lower bands were smaller than those from the corresponding 2A-only targeting vectors. Peptide mapping indicated that the upper bands corresponded to HC polypeptides with residual 2A fragments, while the lower bands represented correctly processed HC polypeptides. Notably, only the RRKR-P2A vector produced an HC polypeptide with the correct size, completely free of residual 2A fragments, as confirmed by peptide mapping (Supplementary Figure S1). However, the display level from this vector was very low, potentially insufficient for antibody library screening.

### 3.2. Point Mutation of P2A in RRKR-P2A for Obtaining Different Display-to-Secretion Ratios

**Figure 3:**
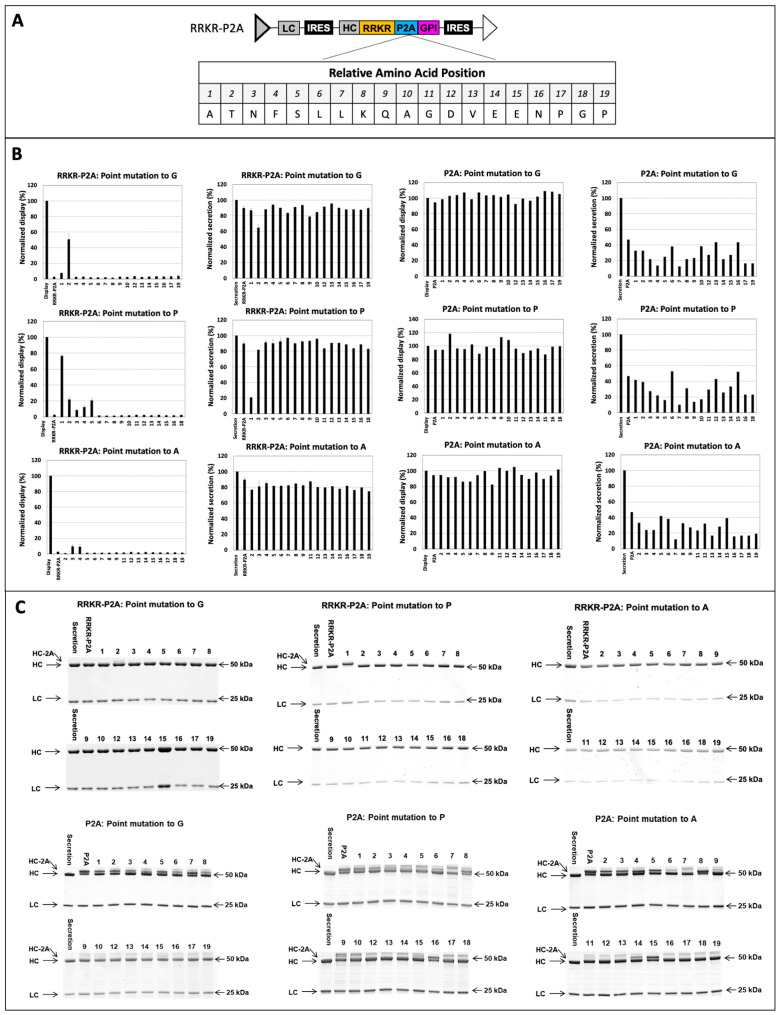
Evaluation of targeting vectors containing P2A variants for antibody display and secretion levels. (**A**) Schematic representation of the targeting vector containing the RRKR-P2A combination and the amino acid sequence of the P2A peptide. Positional scanning mutagenesis was performed within the P2A using substitutions with P, G, and A. Positions 1 to 19 indicate the relative locations of amino acids within the P2A sequence. (**B**) Normalized antibody display and secretion levels in stable pools. These pools were generated by co-transfecting CHO master cells with each targeting vector containing a P2A variant and a helper vector expressing FLPe recombinase, followed by selection in a puromycin-containing medium. Control cell pools were established using the display vector and secretion vector for display and secretion benchmarks, respectively. Stable cell pools were stained with FITC-conjugated IgG specific to the heavy chain of surface-displayed antibodies. Normalized display levels (%) were calculated as the FITC-HC mean fluorescence intensity relative to the display control pool. A 5-day batch culture was performed, and normalized secretion levels (%) were determined as qMab relative to the secretion control pool. (**C**) SDS-PAGE analysis of secreted antibodies under reducing conditions.

To achieve different display-to-secretion ratios using the RRKR-P2A vector, we introduced point mutations in P2A, substituting each amino acid with amino acid G, P, or A (Figure 3). G and P influence secondary structure differently—G increases flexibility, while P imposes conformational constraints. A, on the other hand, has a minimal impact on secondary structure. The mutated targeting vectors were assessed for surface display and antibody secretion as described previously. Nine-point mutations—A1G, T2G, A1P, T2P, N3P, F4P, S5P, N3A, and F4A—enhanced display levels compared to the wild-type RRKR-P2A vector. The extent of the increase varies depending on the mutation. Notably, A1P and T2G mutations raised display levels to 80% and 50% of the display control vector, respectively. Correspondingly, secretion levels from these vectors dropped to 20% and 60% of the secretion control vector, respectively. The HC polypeptides secreted from the A1P vector were larger than those from the secretion control vector. The T2G vector produced two HC bands. Peptide mapping revealed that most HC species from A1P and T2G represented HC polypeptides attached to P2A residues, while a minor proportion retained P2A-GPI residues (Supplementary Figure S1). For the other seven mutations, display levels ranged from 9% to 20% of the display vector, and secretion levels were slightly reduced compared to the secretion vector. Secreted HC polypeptides from these vectors exhibited the correct size on SDS PAGE. However, peptide mapping indicated a small proportion of species retained residual 2A or 2A-GPI fragments (Supplementary Figure S1).

Interestingly, only mutations within the first five amino acids of P2A in the RRKR-P2A vectors increased display levels. Previous studies suggest that mutations at other positions also dramatically reduce 2A peptide cleavage efficiency [44,62]. To explore whether mutations outside the first five amino acids affect P2A cleavage efficiency, one set of targeting vectors containing mutated P2A sequences without mFCS was tested. Analysis of these P2A-only vectors revealed that mutations both within and outside the first five amino acids decreased secretion levels compared to the wild-type P2A vector, likely due to reduced P2A cleavage efficiency. Similarly to the wild-type P2A vector, HC polypeptides expressed from the mutated P2A exhibited two bands. However, these mutations did not significantly further increase display levels compared to the display control vector, suggesting that the number of antibodies displayed on the cell surface had reached saturation. One possible explanation for the lack of increased display levels in mutations outside the first five amino acids for the RRKR-P2A vectors is that these mutations affected only P2A cleavage efficiency. Antibody secretion still occurred via cleavage at RRKR, resulting in the removal of both P2A and GPI from HC. In contrast, the nine mutations within the first five amino acids that increased display levels likely influenced cleavage efficiencies at both RRKR and P2A sites.

### 3.3. Engineering Furin Cleavage Sequence (FCS) for Obtaining Different Display-to-Secretion Ratios

**Figure 4:**
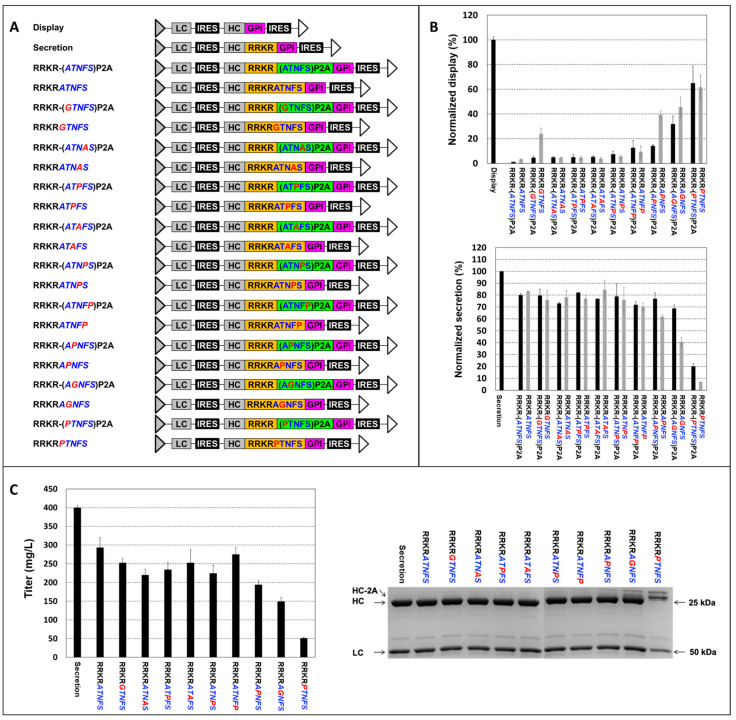
Evaluation of targeting vectors with furin cleavage sequence (FCS) variants or RRKR-P2A combinations for antibody display and secretion levels. (**A**) Schematic representation of targeting vectors incorporating either nine-amino-acid FCS variants or RRKR-P2A combinations. Amino acids highlighted in blue represent the original ATNFS sequence, while those in red indicate the mutations. (**B**) Normalized antibody display and secretion levels relative to control cell pools. Stable pools were generated by co-transfecting CHO master cells with a specified targeting vector and a helper vector expressing FLPe recombinase, followed by selection in a puromycin-containing medium. Display and secretion control pools were established using the display vector and secretion vector, respectively. Flow cytometry analysis was performed on cells stained with FITC-conjugated IgG specific to the heavy chain (FITC-HC) of surface-displayed antibodies. Normalized display levels (%) were calculated as the FITC-HC mean fluorescence intensity relative to the display control pool. Normalized secretion levels (%) were determined based on the qMab values of fed-batch cultures, relative to the secretion control pool. (**C**) Titers of secreted antibodies on day 12 of fed-batch cultures and SDS-PAGE analysis of secreted antibodies under reducing conditions. Each point in panels (**B**,**C**) represents the average and standard deviation of measurements from two independent stable pools.

Previous studies indicate that the five amino acids downstream of the mFCS play a critical role in furin cleavage efficiency [45,46]. To examine the impact of the first five amino acids from the wild-type or mutated P2A on furin cleavage, we designed one set of nine amino-acid FCS variants and linked them to the HC and GPI to enable simultaneous antibody display and secretion. These variants incorporated the first five amino acids from the N-terminus of the wild-type and mutated P2A sequences downstream of RRKR. Corresponding RRKR-P2A vectors with the same mutations were included for comparison. The RRKRATNFS vector, incorporating the first five amino acids (italicized) of the wild-type P2A, exhibited low display and high secretion levels, closely resembling the performance of the wild-type RRKR-P2A vector (Figure 4). All targeting vectors containing the nine FCS variants with the first five amino acids of the mutated P2A showed increased display and decreased secretion compared to the wild-type RRKR-P2A vector. The changes in display and secretion levels closely aligned with those observed in the corresponding mutated RRKR-P2A vectors. Notably, two exceptions, RRKRGTNFS and RRKRAPNFS, displayed significantly higher display levels—approximately five-fold and three-fold higher, respectively—than their RRKR-(GTNFS)P2A and RRKR-(APNFS)P2A counterparts. Correspondingly, secretion levels in the RRKRGTNFS and RRKRAPNFS vectors decreased compared to their P2A-containing counterparts, although the reduction was less pronounced than the increase in display levels. These findings suggest that point mutations in P2A predominantly affect cleavage efficiency at RRKR rather than P2A. However, the discrepancies in display and secretion levels for RRKRGTNFS and RRKRAPNFS vectors relative to their P2A-containing counterparts indicate that mutations A1G and T2P may influence cleavage efficiencies at both RRKR and P2A.

Fed-batch cultures of the pools generated using the ten FCS variants were carried out until day 12. Four variants—RRKRPTNFS, RRKRAGNFS, RRKRAPNFS, and RRKRATNFP—produced titers ranging from 50 to 275 mg/L, with titers inversely correlated to their display levels. Interestingly, the remaining variants did not further enhance antibody titers despite reduced display levels. The harvested antibodies were purified using protein A and analyzed via SDS-PAGE under reducing conditions. HC polypeptides expressed from RRKRAGNFS and RRKRPTNFS vectors, which had the highest display levels, exhibited two distinct bands, with the upper bands having higher molecular weights than those of the secretion control HC. Peptide mapping analysis of the RRKRAGNFS sample revealed that a small proportion of HC polypeptides from the top band remained attached to GPI (Supplementary Figure S1). These aberrant species in the medium may have resulted from cell death. In contrast, HC polypeptides from the other seven FCS variants exhibited the correct molecular weight. Peptide mapping analysis of secreted samples from three targeting vectors—RRKRATNFP, RRKRGTNFS, and RRKRAPNFS—with relative display levels of 9.4%, 23.9%, and 39.4%, respectively, confirmed that the HC polypeptides were correctly processed, with both FCS and GPI successfully removed. The remaining targeting vectors, which exhibited relative display levels below 5%, may not be suitable for antibody screening due to their insufficient display levels.

### 3.4. Comparison of Low- and High-Display-Level Vectors for Discriminating Antibodies with Similar Binding Affinities

**Figure 5:**
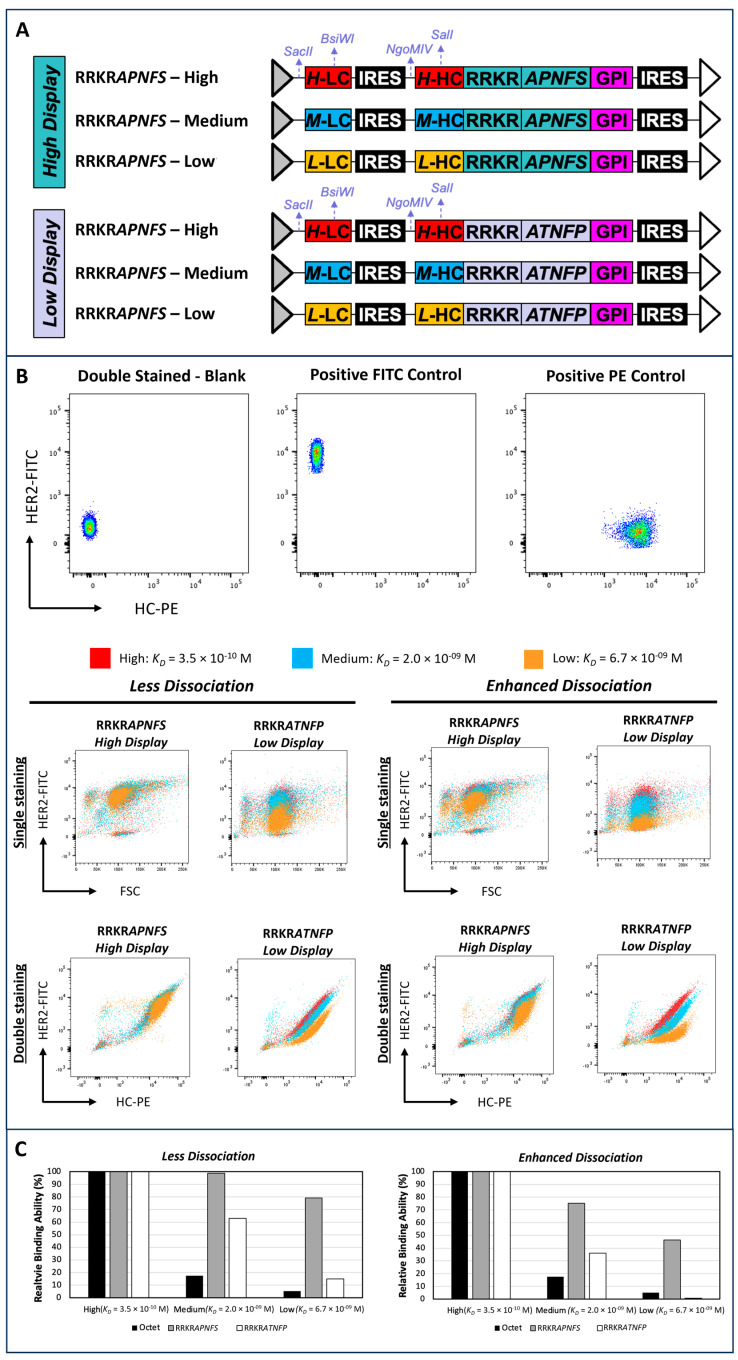
Comparison of low- and high-display level vectors for the discrimination of antibodies with varying binding affinities. (**A**) Schematic representation of high- and low-display level targeting vectors expressing antibodies with high, medium, and low binding affinities. (**B**) Flow cytometry analysis of stable pools expressing different levels of binding affinity antibodies. For the controls, each dot—colored according to the heatmap—represents an individual cell (or ‘event’), with yellow/red indicating high density and blue/green indicating lower density. Different stable pools were generated by co-transfecting CHO master cells with a specified targeting vector and a helper vector expressing FLPe recombinase, followed by selection in puromycin-containing medium. Each stable pool was stained with both FITC-labeled Human Her2/ErbB2 protein (HER2-FITC), R-PE AffiniPure F(ab’)_2_ Fragment Goat Anti-Human IgG, Fc Gamma-specific (HC-PE), using two staining protocols: less or enhanced dissociation phase. (**C**) Comparison of relative binding affinities determined by Octet^®^ analysis and flow cytometry of cells stained with a less or enhanced dissociation phase. The relative binding affinity was normalized to the high-affinity antibody measured under the same method and conditions. Octet^®^ (■), RRKRAPNFS (■), and RRKRATNFP (□).

To evaluate the impact of display levels on the ability to discriminate cells expressing antibodies with close binding affinities, we generated two sets of stable pools using two targeting vectors –RRKRATNFP vector exhibiting display levels of 9.4% (low-display) and RRKRAPNFS vector exhibiting display levels of 39.4% (high-display) relative to the display control, respectively. Each set of stable pools expressed three antibodies against human epidermal growth factor receptor 2 (HER2) antigen with high (KD = 3.5 × 10^−10^ M), medium (KD = 2.0 × 10^−9^ M), and low (KD = 6.7 × 10^−9^ M) binding affinities (Figure 5A). The binding affinities of these antibodies were quantified by Octet^®^ Bio-Layer Interferometry assay. Each stable pool was either single-stained with HER2 antigen labeled with FITC (HER2-FITC) or double-stained with HER2-FITC and R-phycoerythrin (R-PE)-conjugated IgG specific to the heavy chain (HC-PE) staining reagents. Optimal concentrations for each staining reagent, yielding a low signal-to-noise ratio and improved discrimination between cells expressing different antibodies, were identified through titration curves.

The non-transfected cells with double staining served as the blank control, while a stable pool generated using the low-display vector expressing the high-affinity antibody, singly stained with either HER2-FITC or HC-PE, served as the positive control (Figure 5B). Single-staining with HER2-FITC failed to differentiate between the three high-display vector-generated pools expressing high, medium, and low-affinity antibodies (Figure 5B, Less Dissociation). For the three low-display-vector-generated pools, those expressing low and medium binding affinities exhibited distinct levels of fluorescence intensity, but high- and medium-binding affinity pools showed significant overlap. Under double-staining conditions, high-display vector-generated pools continued to exhibit poor discrimination between populations, whereas low-display vector-generated pools demonstrated clear separation between low- and medium-affinity pools and marginal discrimination between medium- and high-affinity pools. Both high-display and low-display vector-generated pools showed overestimated relative affinities compared to measurements obtained using Octet^®^. A better correlation was observed between the Octet^®^ measurements and flow cytometry results for low-display vector-generated pools than for high-display vector-generated pools (Figure 5C, Less Dissociation).

The flow cytometry approach measures binding affinity by incubating cells with staining reagents for 30 min, followed by a brief washing step and immediate analysis. The flow cytometry approach measures binding affinity by incubating cells with staining reagents for 30 min, followed by a brief washing step and immediate analysis. Antibodies with high binding affinity typically exhibit rapid association and slow dissociation. However, this slow dissociation may limit the sensitivity of flow cytometry in distinguishing antibodies with similar high affinities. To improve the resolution of the assay, we modified the staining protocol to enhance dissociation, thereby increasing the sensitivity of flow cytometry in differentiating closely related high-affinity antibodies. This was achieved by incubating cells in the washing buffer at 37 °C for 15 min following antigen staining. Various parameters were tested, including multiple rounds of washing, different incubation times, and larger incubation volumes, to optimize dissociation conditions. Incubating cells at 37 °C for 15 min after the first wash step was found to be most effective, consistent with a previous study that reported enhanced antibody dissociation kinetics at this temperature [63].

Enhancing the dissociation phase in the staining protocol improved the ability to differentiate cell populations (Figure 5B, Enhanced Dissociation). For high-display pools, single-staining could distinguish cells displaying low-binding antibodies from those with high or medium-binding antibodies, though high- and medium-binding populations remained indistinguishable. For low-display pools, discrimination between high- and medium-affinity populations improved slightly, though significant overlap persisted. Under double-staining conditions, high-display pools were still unable to distinguish between high- and medium-binding affinities. In contrast, low-display pools clearly separated cell populations expressing high, medium, and low binding affinities. Supporting this observation, the relative binding affinity of cells stained using the enhanced dissociation protocol for low-display pools closely reflected actual antibody binding kinetics as measured by Octet^®^ (Figure 5C, Enhanced Dissociation). Meanwhile, high-display pools continued to overestimate binding affinities, yielding values several-fold higher than the actual measurements.

### 3.5. Evaluation of Simultaneous Display and Secretion Platform for Sorting Sensitivity

To assess the sensitivity of the simultaneous display and secretion platform to isolate cells expressing high-affinity antibodies from a mixed population via fluorescence-activated cell sorting (FACS), the three low-display vector-generated pools expressing high-, medium-, and low-affinity antibodies were mixed in a 1:1:1 ratio. The mixed cell pool was double-stained with HER2-FITC and HC-PE using the modified staining protocol, which enhanced the dissociation step. The low-display pool expressing the high-affinity antibody was included as a positive control to establish gating parameters. Flow cytometry analysis of the mixed pool revealed three clearly separated populations with similar ratios. Cells within the top 1% gate were sorted and double-stained, showing fluorescence intensities comparable to those of the high-affinity control pool. Sequencing of the LC and HC cDNA from cells in the sorted pools confirmed they were expressing the high-affinity antibody (Supplementary Figure S2).

**Figure 6:**
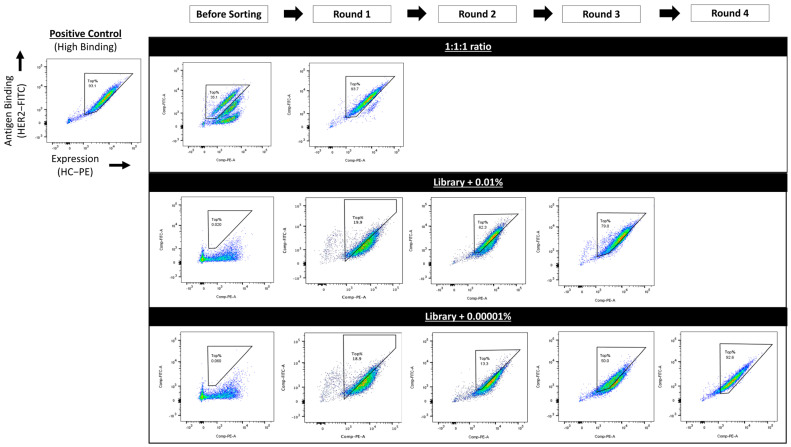
Sorting of stable cell pools spiked with varying percentages of cells expressing high-binding antibodies. Fluorescence-activated cell sorting (FACS) panels were configured with two parameter axes: (1) antigen binding using FITC-labeled Human Her2 (HER2-FITC) and (2) surface expression using R-PE AffiniPure F(ab’)_2_ Fragment Goat Anti-Human IgG, Fc Gamma-specific (HC-PE). Each dot—colored according to the heatmap—represents an individual cell (or ‘event’), with yel-low/red indicating high density and blue/green indicating lower density. A positive control gate, denoted as “Top%”, was established based on the stable pool expressing high-binding antibodies. This gate was consistently applied across repeated rounds of FACS to ensure uniformity in sorting criteria.

To further evaluate the platform’s capability for screening antibody libraries to identify high-affinity binders, a humanized antibody library was constructed. This library was generated by shuffling the human antibody germline frameworks and complementarity-determining regions (CDRs) of the mouse anti-HER2 4D5 antibody, following a previously described method [61]. These humanized antibodies were cloned into the low-display RRKRATNFP vector and co-transfected with an FLPe-expressing helper vector into the CHO master clone, followed by puromycin selection to create a stably transfected cell pool. Cells from the high-affinity control pool were spiked into this newly generated CHO cell pool expressing the humanized antibody library at ratios of 0.01% and 0.0001%. The mixed cell pools were double-stained using the same above-described protocol and subjected to repeated rounds of FACS, selecting the top 1% of high HER2 binders in each round. Under the 0.01% spiking condition, cells expressing antibodies with binding affinities similar to the high-affinity control pool were enriched after three rounds of sorting. For the 0.0001% spiking condition, four rounds of sorting were required to obtain an enriched pool expressing antibodies with affinities similar to the control pool (Figure 6). Sequencing of the LC and HC cDNA in cells from the enriched pools confirmed that the enriched pools were expressing the high-affinity antibody (Supplementary Figure S2).

## 4. Discussion

Previous studies utilized either mFCS or 2A peptide alone between HC and a membrane anchor to achieve simultaneous antibody display and secretion [43,44]. However, engineering mFCS to achieve different display-to-secretion ratios is challenging due to its highly conserved sequences. Meanwhile, the use of 2A peptides resulted in secreted antibodies with attached 2A residues. To address this limitation, we explored the mFCS-2A peptide combinations between HC and a GPI membrane anchor to enable simultaneous antibody display and secretion. The combinations of mFCS (RRKR) with four 2A peptides—P2A, F2A, E2A, and T2A—were evaluated. As anticipated, all vectors linking HC and GPI with these 2A peptides alone produced secreted antibodies containing 2A residues (Figure 2). Notably, the RRKR-P2A vector was the only construct that produced secreted antibodies without attached 2A residues. In contrast, secreted products from RRKR-F2A, RRKR-T2A, and RRKR-E2A vectors contained a mix of HC polypeptides with and without 2A residues (Figure 2 and Supplementary Figure S1).

The 2A peptides facilitate polypeptide cleavage through ribosomal skipping during translation, whereas furin cleavage occurs in the Golgi post-translation. When the gene encoding HC-RRKR-2A-GPI is expressed, the resulting HC polypeptides exist in two forms, HC-RRKR-2A and HC-RRKR-2A-GPI, depending on whether “self-cleavage” occurs at the 2A peptide (Figure 1B). Upon entering the Golgi, cleavage at the RRKR site in both HC-RRKR-2A and HC-RRKR-2A-GPI results in secreted antibodies lacking the 2A and 2A-GPI residues. If furin cleavage does not occur, two antibody forms are produced: one with 2A-GPI attached, displayed on the cell surface, and the other as secreted antibodies with attached 2A residues. The levels of display and the abundance of secreted antibodies, with or without 2A residues, depend on the cleavage efficiency of furin at the RRKR site and the “self-cleavage” efficiency of the 2A peptides.

Among the four RRKR-2A peptide combinations, previous studies have demonstrated that P2A has the lowest cleavage efficiency, E2A and F2A exhibit moderate cleavage efficiencies, and T2A has the highest [47]. In relation to furin cleavage efficiency, the conserved cleavage sequence is described as RP6–XP5–RP4–XP3–(K/R)P2–RP1–XP1’–XP2’–XP3’–XP4’–XP5’. Although not highly conserved, the five amino acids downstream of the core sequence, R-X-K/R-R, also significantly influence furin cleavage efficiency. S, A, F and G at P1’; L, V, A, and S at P2’; S, D, G, P, and E at P3’; A, Q, S, and T at P4’; and A, G, E and L at P5’ are highly prevalent residues in natural FCS [45,46]. The first five amino acids of P2A, F2A, T2A, and E2A are ATNFS, APVKQ, EGRGS, and QCTNY, respectively. Our data (Figure 4), combined with the prevalence of specific amino acids at positions P1’ to P5’ as reported in previous studies [45,46], suggest that positioning ATNFS downstream of RRKR enhances furin cleavage efficiency, while sequences such as APVKQ, EGRGS, and QCTNY may inhibit it. As a result, the dominant HC form expressed from the RRKR-P2A vector in the endoplasmic reticulum is HC-RRKR-P2A-GPI, with HC-RRKR-P2A as the minor form due to P2A’s low cleavage efficiency. Upon Golgi entry, efficient cleavage at RRKR in both HC-RRKR-P2A and HC-RRKR-P2A-GPI results in high levels of secreted antibodies without 2A residues and low levels of cell surface display. For the RRKR-F2A and RRKR-E2A vectors, HC-RRKR-2A and HC-RRKR-2A-GPI are both dominant due to moderate 2A cleavage efficiency. Low furin cleavage efficiency at RRKR in the Golgi results in incomplete processing of both HC-RRKR-2A and HC-RRKR-2A-GPI, leading to the secretion of antibodies in two forms, with and without 2A residues, and relatively higher display levels compared to the RRKR-P2A vector. In the RRKR-T2A vector, HC-RRKR-2A is the dominant form due to T2A’s high cleavage efficiency, while HC-RRKR-T2A-GPI is the minor form. However, low furin cleavage efficiency in the Golgi leads to high secretion levels of antibodies in both forms, with and without 2A residues, and lower display levels. Further studies showed that mutating P2A in the mFCS-P2A vector increased the antibody display-to-secretion ratio by inhibiting furin cleavage, resulting in incomplete processing of HC-RRKR-P2A-GPI. However, this also led to the secretion of antibodies with 2A residues attached. These findings indicate that achieving both high antibody display levels and secretion of only the correct product is not feasible with the RRKR-2A strategy.

A previous study demonstrated simultaneous antibody display and secretion by linking the HC to the PDGFR membrane anchor via an mFCS sequence, RIRR [43]. However, in our study, when HC was linked to a GPI membrane anchor using a similar mFCS sequence, RRKR, antibodies were expressed exclusively in the membrane-bound form, with no detectable secretion (Figure 1). Given that RIRR and RRKR are expected to have comparable furin cleavage efficiencies, the absence of secretion in our study is likely attributable to the use of different membrane anchors rather than differences in the mFCS sequences. The first five amino acids of GPI are P, N, K, G, and S, whereas those of PDGFR are A, V, G, Q, and D. Notably, P at P1’ and N at P2’ are rarely observed in natural FCS, whereas A at P1’ and V at P2’ are highly prevalent in FCS with high furin cleavage efficiency [45,46]. This difference likely explains the lack of secretion in the RRKR vector, as furin cleavage is inhibited by the P and/or N residues at P1’ and P2’ in GPI. This hypothesis is further supported by data showing that the RRKR-P2A vector exhibited very low display levels, while mutating the first amino acid of P2A from A to P significantly increased display levels (Figure 3). Similarly, the RRKRATNFS variant showed low display levels, whereas the RRKRPTNFS variant exhibited high display levels (Figure 4). These results suggest that P at P1’ strongly inhibits furin cleavage efficiency.

Further analysis of the ten nine-amino-acid FCS variants, incorporating the first five amino acids from the N-terminus of the wild-type and mutated P2A sequences downstream of RRKR, provided deeper insights into how specific amino acids and their positions affect furin cleavage efficiency (Figure 4). For instance, P at positions P2’ to P5’ inhibited furin cleavage, although the effect was less pronounced than at P1’. G at P2’ strongly inhibited cleavage, while its effect at P1’ was weaker. A at P3’ to P4’ also inhibited cleavage but had no impact when located elsewhere. The amino acid composition at specific positions in FCS variants with high furin cleavage efficiency aligned with the prevalence of highly conserved amino acids at positions P1’ to P5’, as reported in previous studies [45,46]. This suggests that incorporating highly conserved amino acids at each position is beneficial for enhancing furin cleavage efficiency. These findings are pivotal for designing optimized FCS variants, enabling precise control over antibody display-to-secretion ratios and supporting diverse applications.

The ten nine-amino-acid FCS variants identified in this study modulated antibody display-to-secretion ratios ranging from 3.1% to 61.6%. Since the total amount of antibodies expressed by a single cell is fixed, increasing the amount of antibodies displayed on the cell surface will lead to lower secretion levels (Figure 4). Higher secretion levels are preferable for reducing cost-of-goods, making the optimal display-to-secretion ratio one that minimizes display levels while still enabling effective antibody library screening. In addition, excessively high display levels can saturate the cell surface, diminishing the platform’s ability to differentiate between cells expressing antibodies with similar binding affinities [38]. By comparing two targeting vectors with display levels of 9% and 39% relative to a control display vector, we demonstrated that lower display levels more effectively distinguish cells expressing antibodies with high and closely related binding affinities. While further reducing display levels could enhance the ability to discriminate between antibodies with different affinities, excessively low display levels can result in poor signal-to-noise ratios, negatively impacting platform performance. Low display levels may not be ideal for all applications. For example, in antibody discovery using large libraries, where positive antibodies are rare and often exhibit low binding affinities, higher display levels can improve surface staining signals and increase sensitivity for detecting positive hits.

Our simultaneous antibody display and secretion platform demonstrated high sensitivity for isolating high-affinity antibody-expressing cells, while achieving antibody production levels exceeding 200 mg/L in fed-batch cultures. We validated its sensitivity by successfully isolating cells expressing a known high-affinity antibody from a pool of humanized antibody library variants, with the cells expressing the known antibody spiked at only 0.0001%. However, no antibodies with higher binding affinity than the control antibody were identified. This outcome is likely due to the limited size of the humanized antibody library integrated into the CHO master cells. The humanized antibody library we constructed contains approximately 10 million variants, but the FLP recombinase system used has an integration efficiency of around 1% (unpublished data). As a result, the transfection of 10 million CHO master cells results in the expression of only about 1 × 10^5^ unique antibodies. This limitation in library size is a common drawback of mammalian cell display systems. Enhancing targeted integration efficiency through more effective recombinase systems, such as BxB1, could address this issue [64,65,66,67]. Additional strategies include enriching libraries via phage display before transitioning to mammalian display, utilizing immunized libraries, or designing high-quality libraries using artificial intelligence [6,55].

Another critical parameter for our platform is secretion level. While the achieved production level of over 200 mg/L is sufficient for many preclinical developability and functional studies—such as assessing antibody stability, aggregation, and some animal studies—applications like toxicity studies in animals and clinical trials typically require titers in the range of several grams per liter. To expand the platform’s applications, strategies for further enhancing expression levels are necessary. Genome-wide screening to identify more active genomic sites could significantly boost expression. Optimizing the targeting vector is another approach. The current vector employs EMCV IRES to drive HC expression, which has lower translation efficiency compared to cap-dependent translation. Replacing EMCV IRES with 2A peptides for co-expression of LC and HC could substantially enhance antibody expression levels [68].

## Data Availability

All data generated or analyzed during this study are included in this published article and Supplementary Materials.

## Supplementary Materials

The following supporting information can be downloaded at https://www.mdpi.com/article/10.3390/antib14020038/s1, Supplementary Figure S1: Peptide mapping analysis of HC polypeptides expressed from various targeting vectors. Supplementary Figure S2: Validation of sorted clones. Supplementary Table S1: PCR primer pairs for HC and LC.

## Abbreviations

The following abbreviations are used in this manuscript:

CHO	Chinese hamster ovary
ZZ-PDGFR	Dual Z-domain with PDGFR transmembrane domain
EMCV	Encephalomyocarditis virus
ECSTASY	Enzyme-cleavable surface-tethered all-purpose screening system
E2A	Equine rhinitis A virus 2A peptide
FACS	Fluorescence-activated cell sorting
Fab	Fragment antigen binding
FITC	Fluorescein isothiocyanate
FITC-HC	FITC-conjugated IgG specific to the heavy chain
F2A	Foot-and-mouth disease virus 2A peptide
GPI	Glycophospholipid
HC	Heavy chain
HC-PE	F(ab’)_2_ Fragment Goat Anti-Human IgG, Fc Gamma specific phycoerythrin
HER2	Human epidermal growth factor receptor 2
HER2-FITC	HER2-Fluorescein isothiocyanate
HYGR	Hygromycin resistance
IgG	Immunoglobulin G
IVCD	Integrated viable cell density
IRES	Internal ribosomal entry site
LC	Light chain
MFI	Mean fluorescence intensity
mFCS	Minimal furin cleavage sequence
F3	Mutated FRT3
PFA	Peptide feature area
Pcd	Pg cell^−1^ day^−1^
PDGFR	Platelet-derived growth factor receptor
pA	Polyadenylation signals
P2A	Porcine teschovirus-1 2A peptid
RMCE	Recombinase-mediated cassette exchange
R-PE	R-phycoerythrin
scFv	Single-chain variable fragment
qMab	Specific productivity
(ATG-)Puro	Start codon-deficient puromycin resistance gene
T2A	Thosea asigna virus 2A peptide
VH	Variable Heavy
VL	Variable Light
F	Wild-type FRT
