# Supplementary material for: A Targeted Integration-Based CHO Cell Platform for Simultaneous Antibody Display and Secretion
Source: Antibodies (Basel). 2025 Apr 28;14(2):38. doi: 10.3390/antib14020038 (PMC12101391; doi:10.3390/antib14020038)
Supplement: Supplementary file 1 [file antibodies-14-00038-s001.zip › antibodies-3583290-supplementary.pdf]

Supplementary Figure S1. Peptide mapping analysis of HC polypeptides expressed from various targeting vectors. The intensity of each peptide is indicated as the area in tables.

HC polypeptide expressed from the Secretion targeting vector

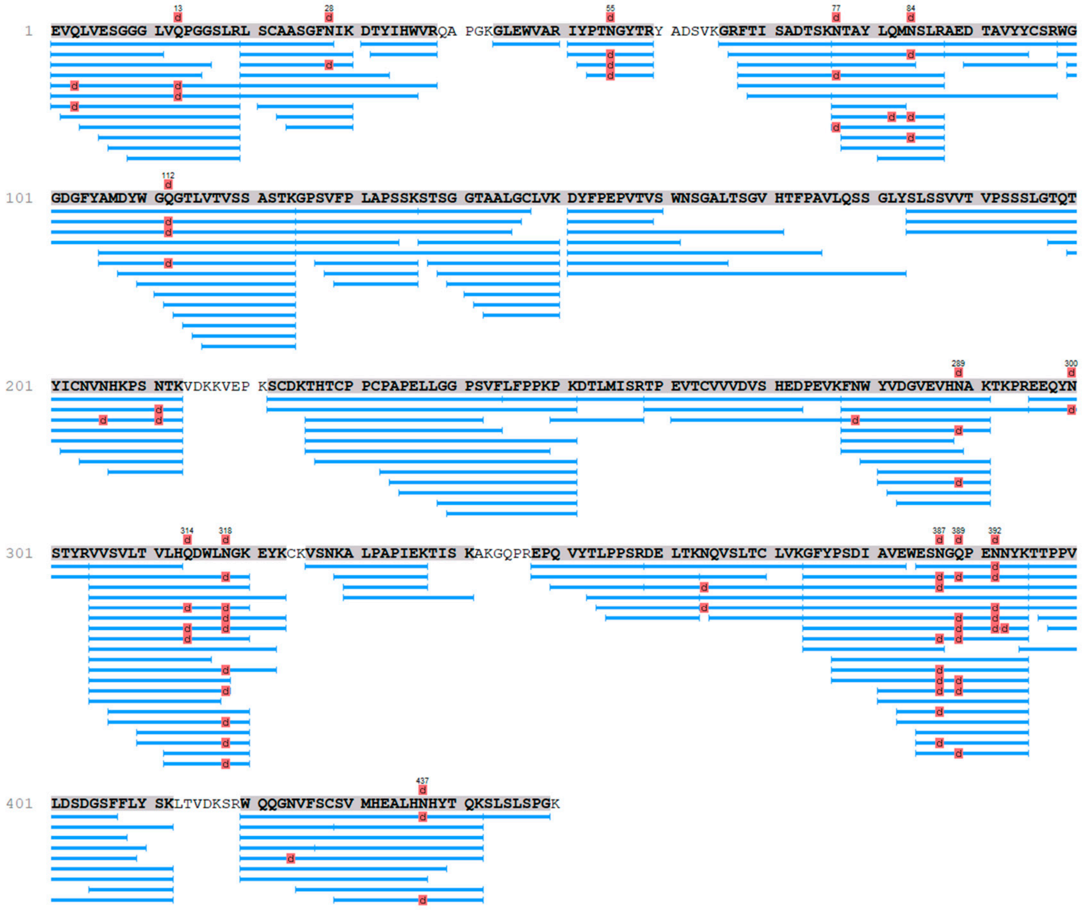

Top band of HC polypeptide expressed from targeting vector P2A

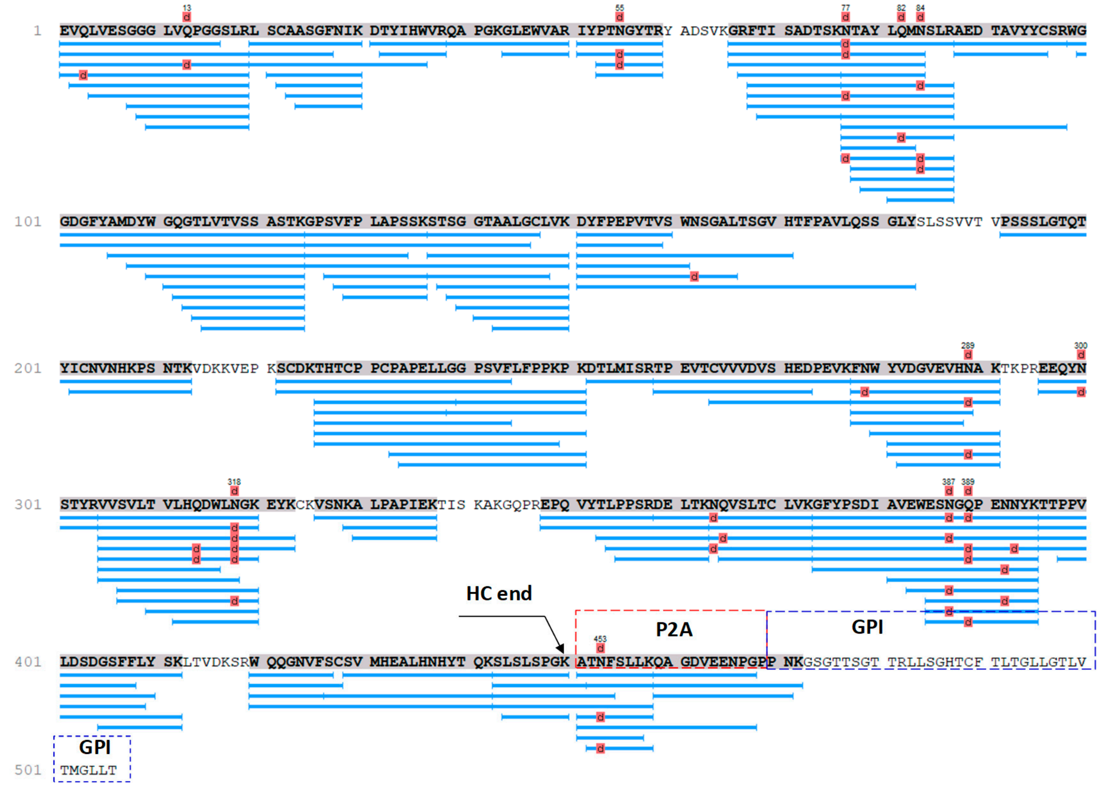

		Peptide	Unique	-10lgP	Mass	Length	ppm	m/z	RT	Area F0P2AWT_A	Scan	#Feature	Start	End
1	☑	K.QAGDVEENPGPPNK.G	•	87.83	1450.6688	14	0.7	726.3422	19.59	9.5E5	2882	1	459	472
2	☑	K.QAGDVEENPGPPN.K	•	66.41	1322.5739	13	-0.6	662.2938	23.98	1.68E5	3588	1	459	471
3	☑	K.QAGDVEENPGPPNKG.S	•	61.87	1507.6902	15	-1.0	754.8516	19.69	0	2929	0	459	473
4	☑	K.QAGDVEENPG.P	•	56.64	1014.4254	10	-0.4	508.2198	22.97	2.05E7	3451	1	459	468
5	☑	A.TN(+.98)FSLLK.Q	•	39.97	822.4487	7	-0.8	412.2313	55.67	2.59E4	9209	1	452	458

Bottom band of HC polypeptide expressed from targeting vector P2A

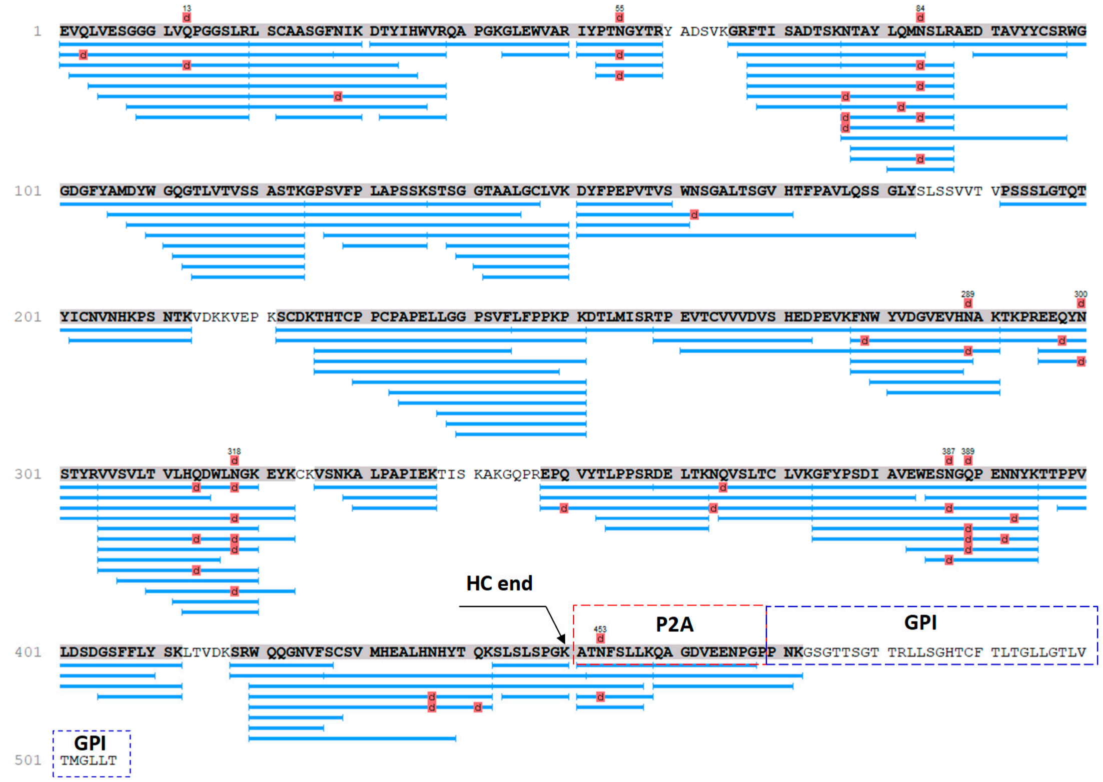

		Peptide	Unique	-10lgP	Mass	Length	ppm	m/z	RT	Area F0P2AWT_B	Scan	#Feature	Start	End
1	☑	K.QAGDVEENPGPPNK.G	•	87.67	1450.6688	14	-0.1	726.3416	19.24	1.85E5	2831	1	459	472
2	☑	K.QAGDVEENPG.P	•	58.11	1014.4254	10	0.6	508.2203	16.82	2.07E6	2613	2	459	468
3	☑	K.QAGDVEENPGPPN.K	•	53.24	1322.5739	13	0.7	662.2947	23.91	6.94E3	3521	1	459	471
4	☑	A.TNFSLLK.Q	•	39.67	821.4647	7	-0.6	411.7394	51.30	3.05E4	8024	1	452	458
5	☑	K.ATN(+.98)FSLLK.Q	•	80.04	893.4858	8	-0.5	447.7499	53.88	4.24E5	8493	2	451	458
6	☑	K.ATNFSLLK.Q	•	78.19	892.5018	8	-1.1	447.2577	51.24	6.89E6	8001	1	451	458
7	☑	K.ATNFSLL.K	•	37.36	764.4069	7	-0.9	383.2104	68.55	1.52E5	11104	1	451	457

Top band of HC polypeptide expressed from targeting vector F2A

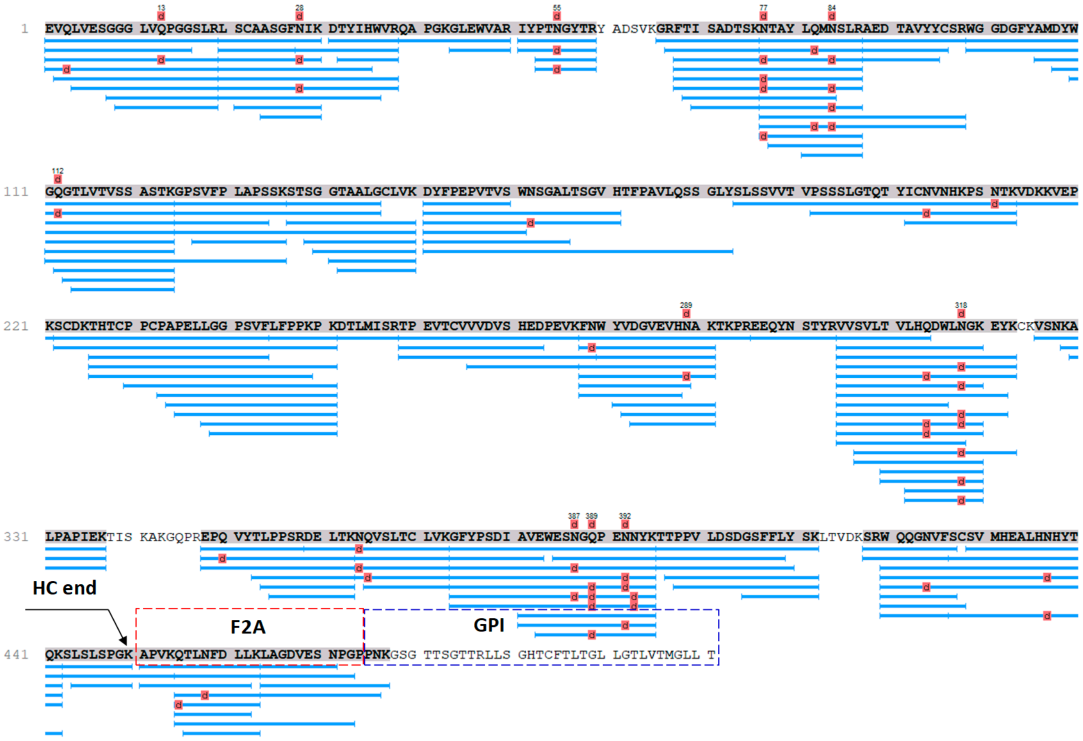

		Peptide	Unique	-10lgP	Mass	Length	ppm	m/z	RT	Area F0F2AWT_A	Scan	#Feature	Start	End
1	☑	K.LAGDVESNPGPPNK.G	•	85.44	1393.6837	14	0.7	697.8496	24.84	7.88E5	3711	1	464	477
2	☑	K.LAGDVESNPGPPN.K	•	70.06	1265.5887	13	-0.2	633.8015	29.92	1.89E5	4525	1	464	476
3	☑	K.LAGDVESNPG.P	•	68.26	957.4403	10	-0.5	479.7272	27.66	3.19E7	4170	5	464	473
4	☑	K.LAGDVESNPGPPNKG.S	•	57.14	1450.7052	15	0.8	726.3605	24.81	0	3735	0	464	478
5	☑	K.LAGDVESNP.G	•	37.16	900.4188	9	0.4	451.2169	25.37	0	3818	0	464	472
6	☑	K.LAGDVESN.P	•	30.56	803.3661	8	-0.1	804.3733	20.37	8.5E4	3117	1	464	471
7	☑	Q.TLNFDLLK.L	•	55.11	962.5436	8	1.1	482.2796	68.60	3.56E4	11342	1	456	463

Bottom band of HC polypeptide expressed from targeting vector F2A

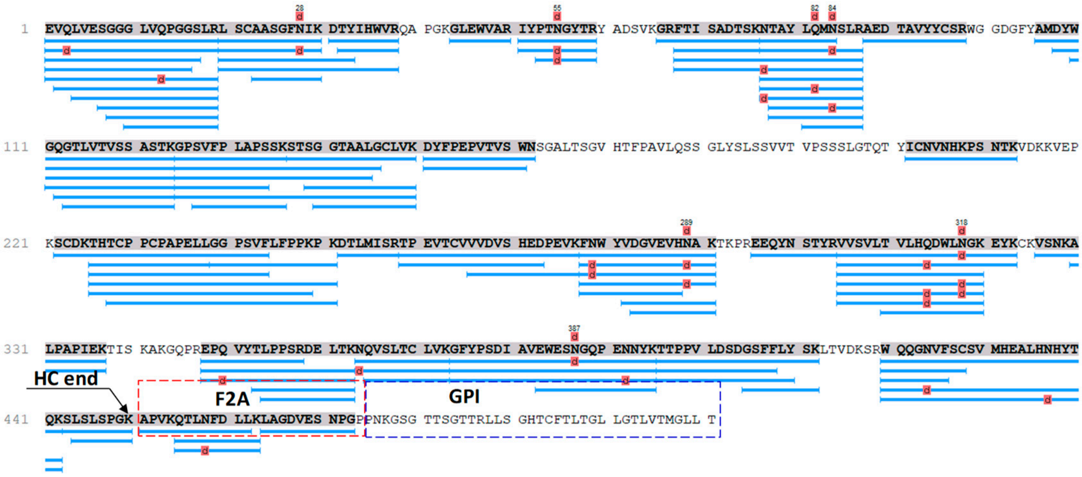

		Peptide	Unique	-10lgP	Mass	Length	ppm	m/z	RT	Area F0F2AWT_B	Scan	#Feature	Start	End
1	☑	K.LAGDVESNPG.P	•	59.37	957.4403	10	-1.3	479.7268	23.68	3.3E5	3157	2	464	473
2	☑	K.LAGDVESNPGPPNK.G	•	59.16	1393.6837	14	-0.8	697.8486	24.65	0	3246	0	464	477
3	☑	K.LAGDVESNPGPPN.K	•	44.89	1265.5887	13	-0.4	633.8014	29.86	0	3895	0	464	476
4		K.LAGDVESNPGPPNKGSGTTSGTTR.L	•	12.22	2299.1040	24	0.8	767.3759	59.95	0	8513		464	487
5	☑	K.QTLNFDLLK.L	•	60.66	1090.6023	9	0.1	546.3085	62.94	1.9E4	9021	2	455	463

HC polypeptide expressed from targeting vector T2A

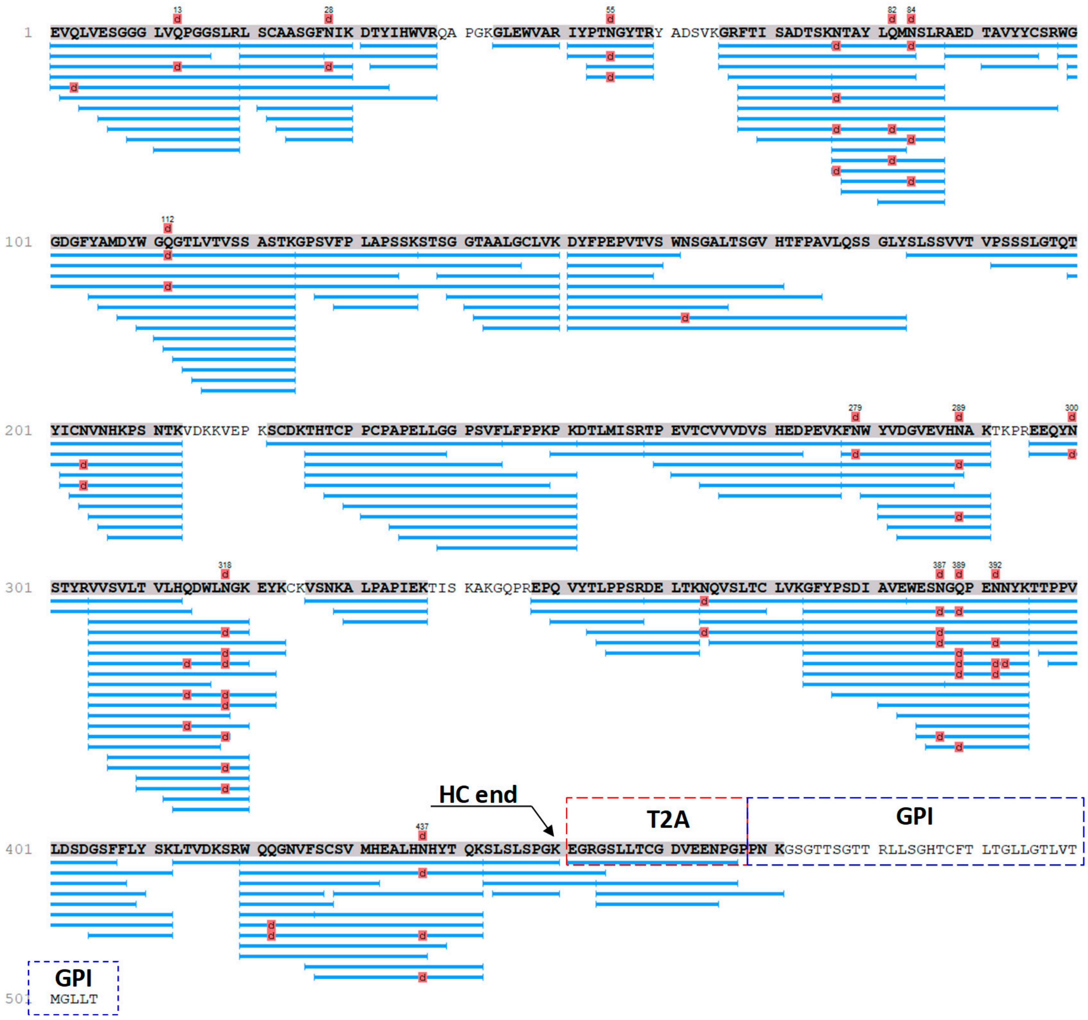

		Peptide	Unique	-10lgP	Mass	Length	ppm	m/z	RT	Area F0T2AWT	Scan	#Feature	Start	End
1	☑	R.GSLLTC(+57.02)GDVEENPGPPNK.G	•	139.09	1882.8730	18	-1.2	942.4427	43.43	3.75E4	7166	1	454	471
2	☑	R.GSLLTC(+57.02)GDVEENPG.P	•	95.15	1446.6296	14	1.6	724.3232	47.14	3.33E7	7804	5	454	467
3	☑	R.GSLLTC(+57.02)GDVEEN.P	•	78.23	1292.5554	12	0.0	647.2850	45.02	5.79E5	7437	2	454	465
[illegible]	[illegible]	[illegible]	[illegible]	[illegible]	[illegible]	[illegible]	[illegible]	[illegible]	[illegible]	[illegible]	[illegible]	[illegible]	[illegible]	[illegible]

Top band of HC polypeptide expressed from targeting vector E2A

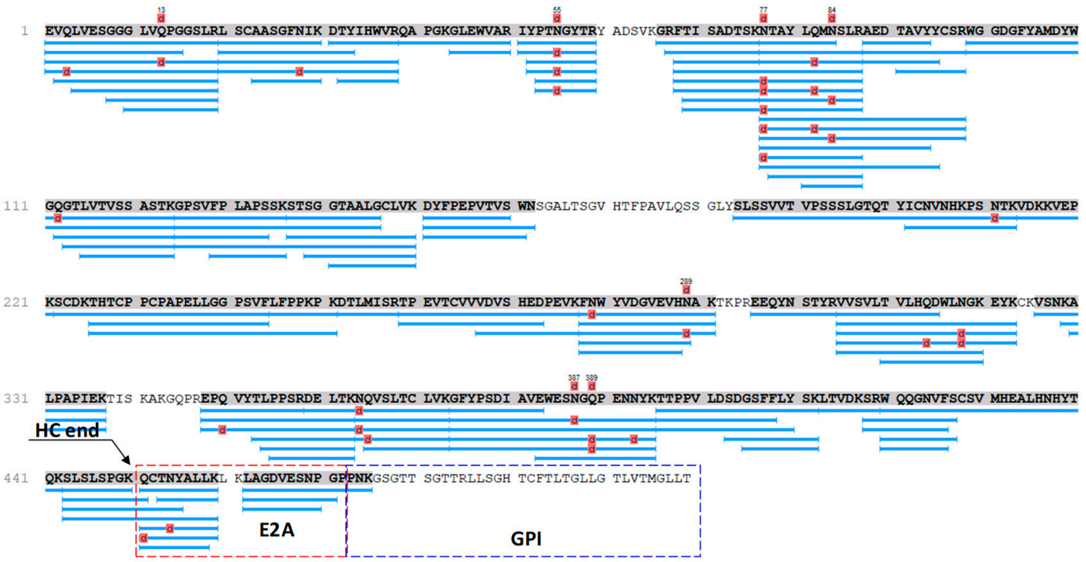

		Peptide	Unique	-10lgP	Mass	Length	ppm	m/z	RT	Area F0E2AWT_A	Scan	#Feature	Start	End
1	☑	K.LAGDVESNPGPPNK.G	•	79.04	1393.6837	14	0.9	697.8498	24.61	1.61E4	3576	1	462	475
2	☑	K.LAGDVESNPG.P	•	77.47	957.4403	10	0.4	479.7276	23.52	2.54E7	3530	1	462	471
3	☑	K.LAGDVESNP.G	•	46.53	900.4188	9	-0.3	451.2166	25.18	0	3670	0	462	470

Bottom band of HC polypeptide expressed from targeting vector E2A

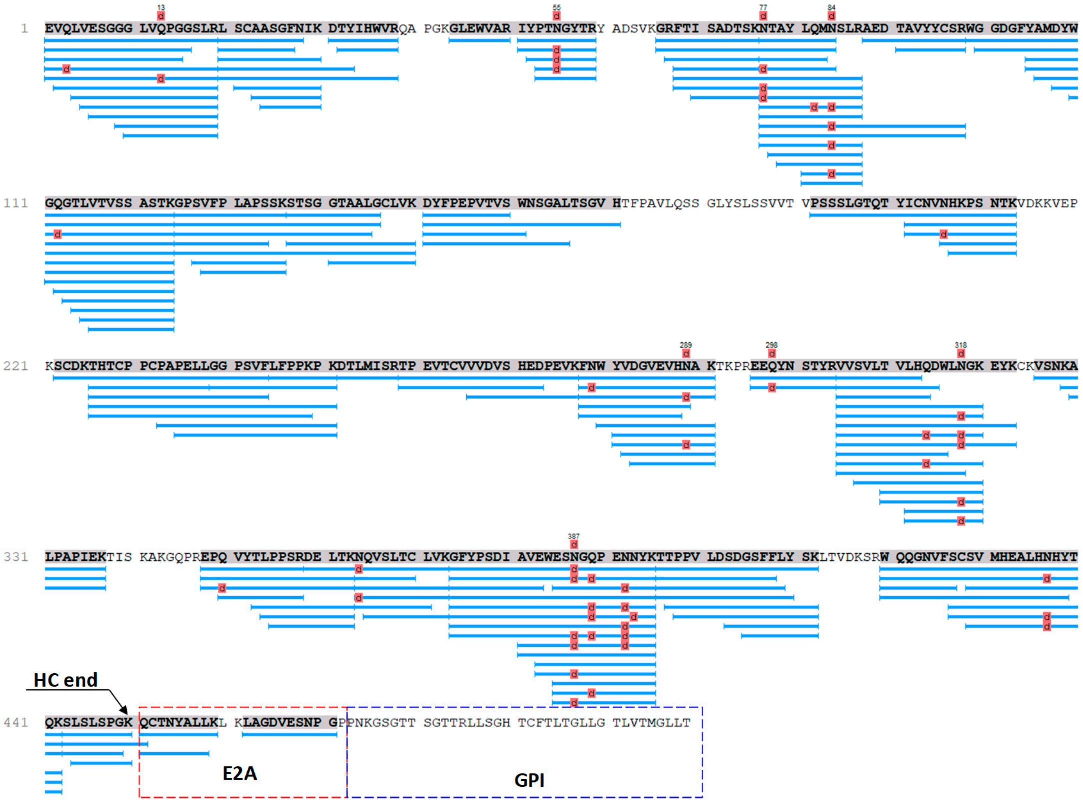

HC polypeptide expressed from targeting vector RRKR-P2A

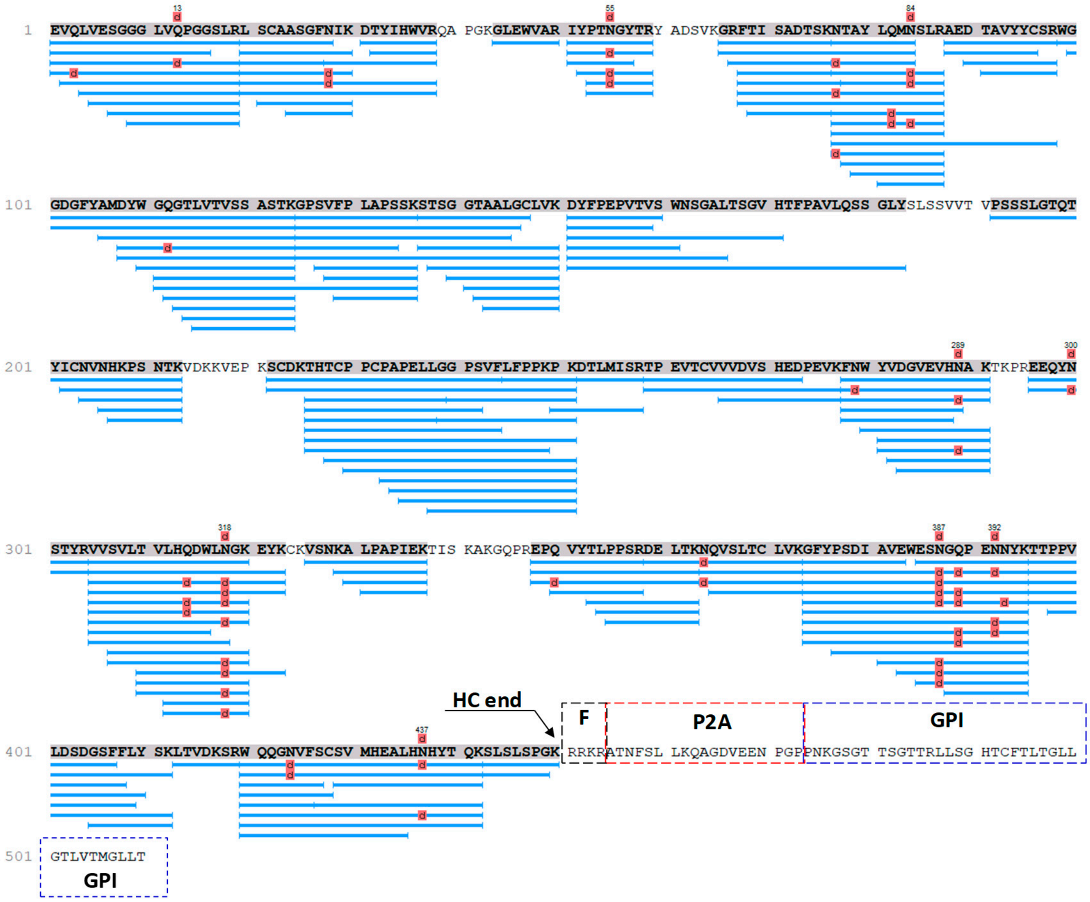

Top band of HC polypeptide expressed from targeting vector RRKR-F2A

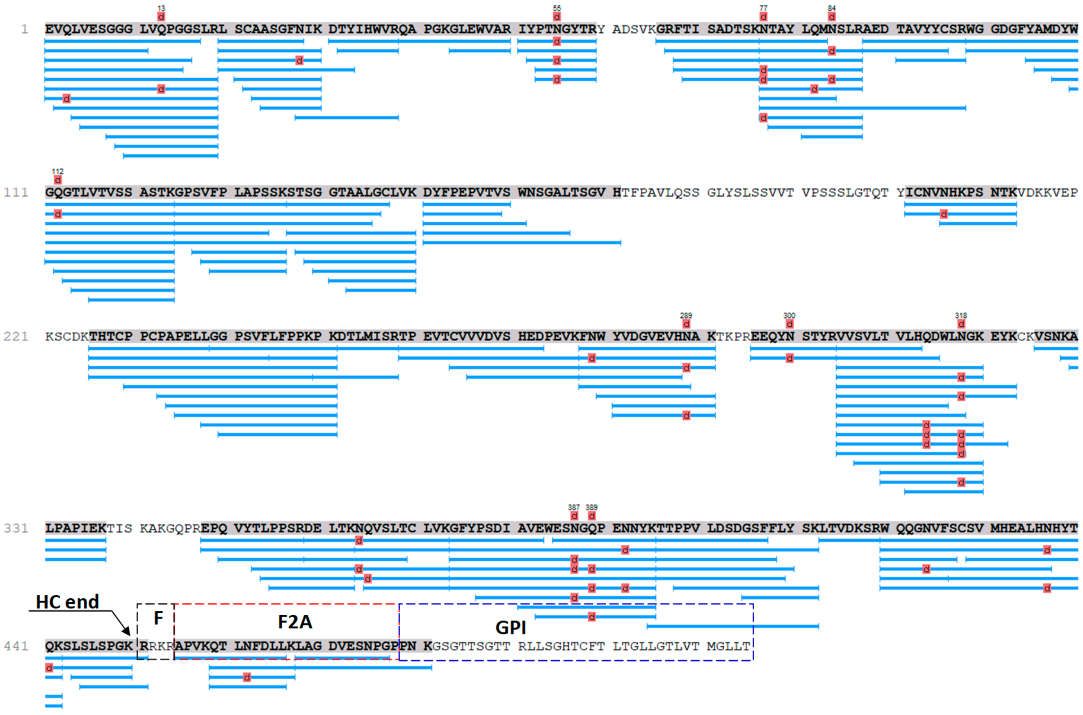

		Peptide	Unique	-10lgP	Mass	Length	ppm	m/z	RT	Area F2AWT_A	Scan	#Feature	Start	End
1	☑	K.LAGDVESNPGPPNK.G	•	89.03	1393.6837	14	-0.4	697.8489	24.86	5.8E4	3894	1	468	481
2	☑	K.LAGDVESNPG.P	•	72.32	957.4403	10	0.5	479.7277	23.85	2.03E6	3721	1	468	477

Bottom band of HC polypeptide expressed from targeting vector RRKR-F2A

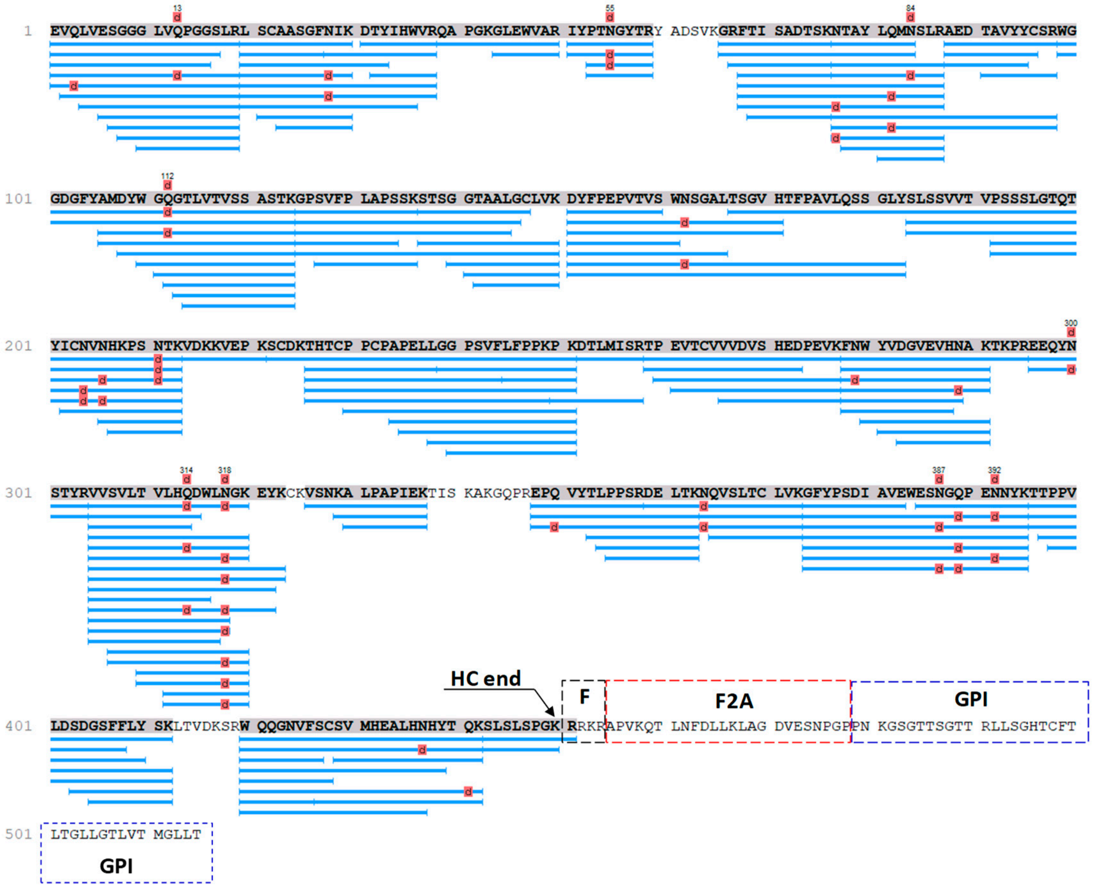

Top band of HC polypeptide expressed from targeting vector RRKR-T2A

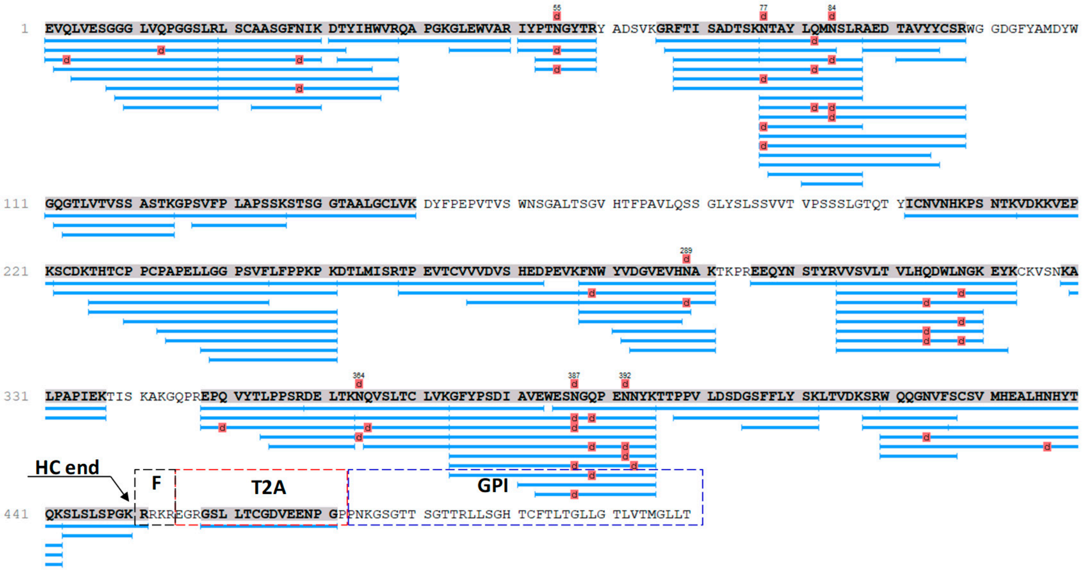

		Peptide	Unique	-10lgP	Mass	Length	ppm	m/z	RT	Area T2AWT_A	Scan	#Feature	Start	End
1	☑	R.GSLLTC(+57.02)GDVEENPG	•	94.29	1446.6296	14	0.2	724.3222	47.49	2.6E6	7208	2	458	471
2		R.GSLLTC(+57.02)GDVEEN(+.98)PG	•	9.01	1447.6136	14	0.5	724.8145	80.75	0	12658		458	471
3		L.SLSPGKR.R	•	23.52	743.4290	7	0.0	372.7217	25.99	8.46E5	3711		445	451
4		S.LSLSPGK.R	•	17.03	700.4119	7	0.1	351.2133	31.90	1.41E4	4646		444	450
5	☑	K.SLSLSPGKR.R	•	58.66	943.5450	9	0.6	472.7801	26.06	3.3E6	3722	2	443	451
6	☑	K.SLSLSPGK.R	•	62.12	787.4440	8	-0.9	394.7289	31.85	9.49E6	4604	2	443	450
[illegible]	[illegible]	[illegible]	[illegible]	[illegible]	[illegible]	[illegible]	[illegible]	[illegible]	[illegible]	[illegible]	[illegible]	[illegible]	[illegible]	[illegible]

Bottom band of HC polypeptide expressed from targeting vector RRKR-T2A

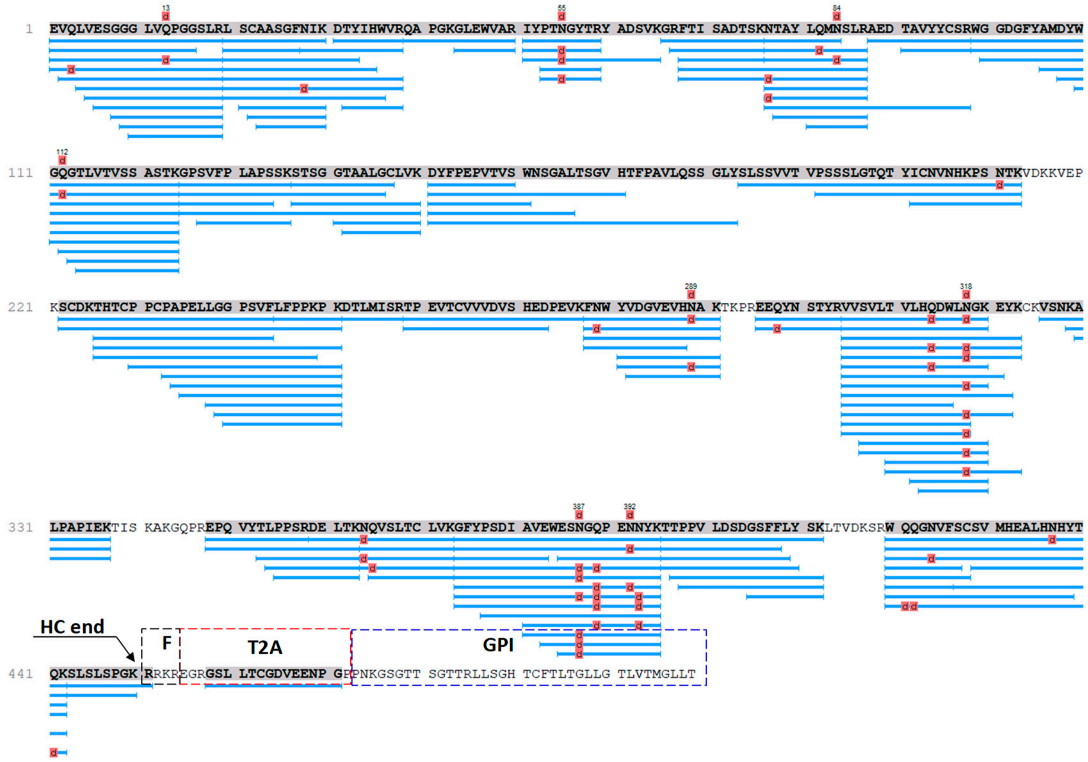

		Peptide	Unique	-10lgP	Mass	Length	ppm	m/z	RT	Area T2AWT_B	Scan	#Feature	Start	End
1	☑	R.GSLLTC(+57.02)GDVEENPG.P	•	90.13	1446.6296	14	0.7	724.3226	47.31	1.33E5	7279	1	458	471
2		G.RGSLLTC(+57.02)GDVEEN(+.98)PGPP...	•	5.42	2039.9581	19	-1.4	680.9924	69.95	0	11354		457	475
3		S.LSLSPGK.R	•	19.20	700.4119	7	-1.1	351.2129	31.78	0	4786		444	450
4	☑	K.SLSLSPGK.R	•	57.67	787.4440	8	-0.4	394.7291	31.77	3.81E6	4850	1	443	450
5	☑	K.SLSLSPGKR.R	•	44.86	943.5450	9	0.0	472.7798	26.02	6.17E4	3910	1	443	451

Top band of HC polypeptide expressed from targeting vector RRKR-E2A

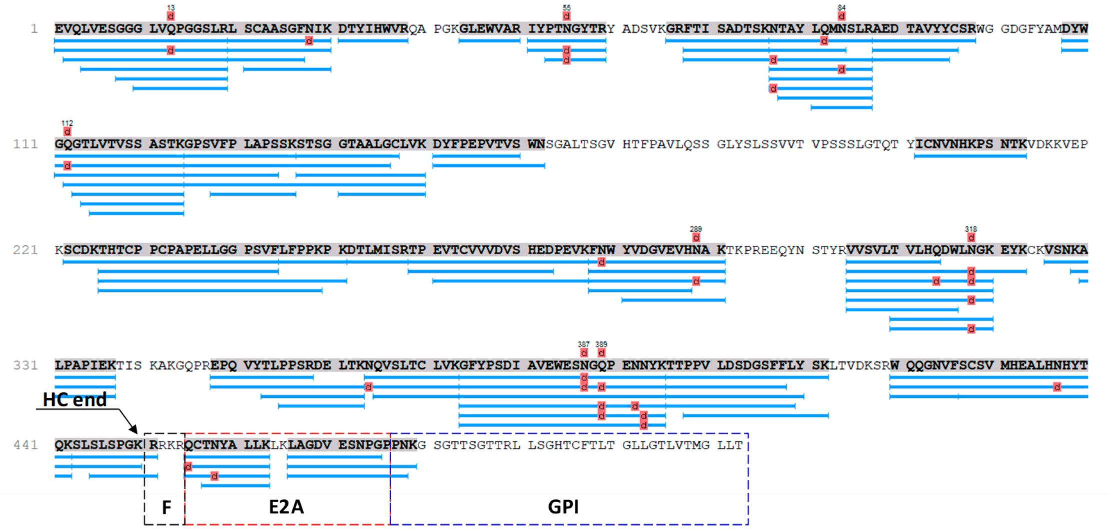

		Peptide	Unique	-10lgP	Mass	Length	ppm	m/z	RT	Area E2AWT_A	Scan	#Feature	Start	End
1		N.PGPPNKGSGTTSGTTR.L	•	9.26	1513.7484	16	-3.9	505.5881	9.97	0	1500		474	489
2	☑	K.LAGDVESNPGPPNK.G	•	100.47	1393.6837	14	-1.1	697.8484	21.95	1.49E5	3186	1	466	479
3	☑	K.LAGDVESNPGPPN.K	•	77.52	1265.5887	13	0.6	633.8020	28.63	5.64E4	4149	1	466	478
4	☑	K.LAGDVESNPG.P	•	74.91	957.4403	10	-0.3	479.7273	24.81	1.53E7	3592	3	466	475

Bottom band of HC polypeptide expressed from targeting vector RRKR-E2A

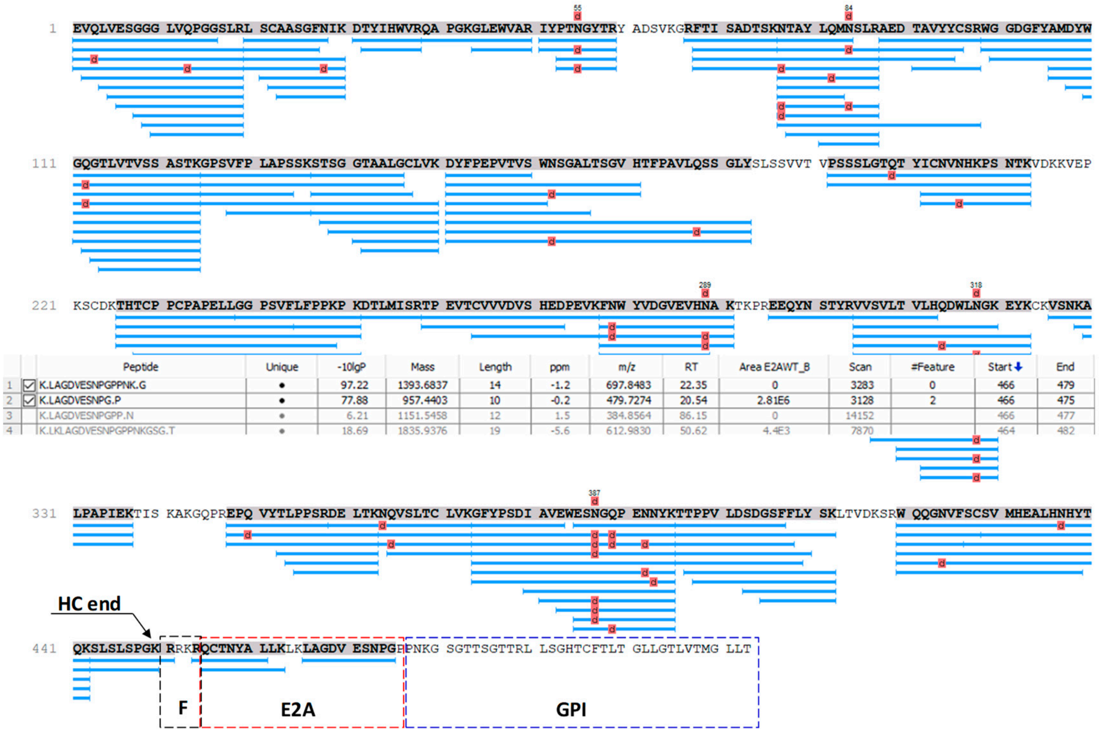

		Peptide	Unique	-10lgP	Mass	Length	ppm	m/z	RT	Area E2AWT_B	Scan	#Feature	Start	End
1	☑	K.LAGDVESNPGPPNK.G	•	97.22	1393.6837	14	-1.2	697.8483	22.35	0	3283	0	466	479
2	☑	K.LAGDVESNPG.P	•	77.88	957.4403	10	-0.2	479.7274	20.54	2.81E6	3128	2	466	475
3		K.LAGDVESNPGPP.N	•	6.21	1151.5458	12	1.5	384.8564	86.15	0	14152		466	477
4		K.LKLAGDVESNPGPPNKGSG.T	•	18.69	1835.9376	19	-5.6	612.9830	50.62	4.4E3	7870		464	482

HC polypeptide expressed from targeting vector RRKR-P2A(A1G)

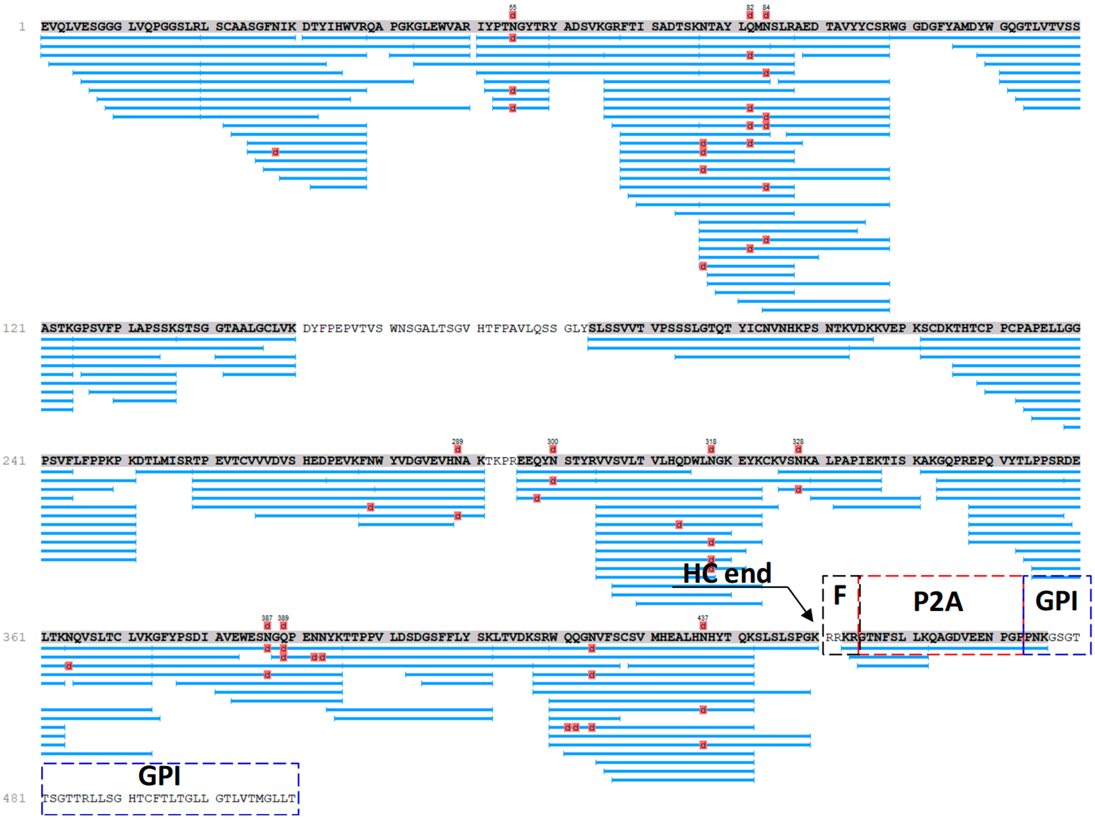

	T.C(+57.02)FTLTGLLGTLVTM(+15.99)GLLT		7.09	1926.0206	18	-1.5	964.0161	67.42	3.98E4	7547		493	510
	G.SGTTSGTTR.L		14.98	866.4094	9	8.7	434.2157	25.72	9.02E3	2407		478	486
	E.ENPGPPNK.G		6.38	851.4137	8	-3.8	852.4177	76.11	1.37E4	8617		469	476
☑	K.QAGDVEENPGPPNK.G		81.39	1450.6688	14	0.3	726.3419	19.96	8.26E4	1707	1	463	476
☑	R.GTNFSLLK.Q	•	73.24	878.4861	8	-0.5	440.2501	47.67	4.87E5	4975	1	455	462
☑	K.RGTNFSLLK.Q	•	62.55	1034.5873	9	0.3	518.3011	40.53	2.48E5	4073	1	454	462
	K.RGTNFSL.L	•	28.36	793.4082	7	0.8	397.7117	40.53	1.32E4	4082		454	460
☑	R.KRGTNFSLLK.Q	•	54.96	1162.6823	10	0.6	388.5683	33.39	5.75E4	3238	1	453	462
☑	K.SLSLSPGK.R		59.80	787.4440	8	0.1	394.7293	29.13	5.52E6	2743	1	443	450
☐	K.SLSLSPG.K		52.91	659.3490	7	-0.4	330.6816	38.17	0	3803	0	443	449

Top band of HC polypeptide expressed from targeting vector RRKR-P2A(T2G)

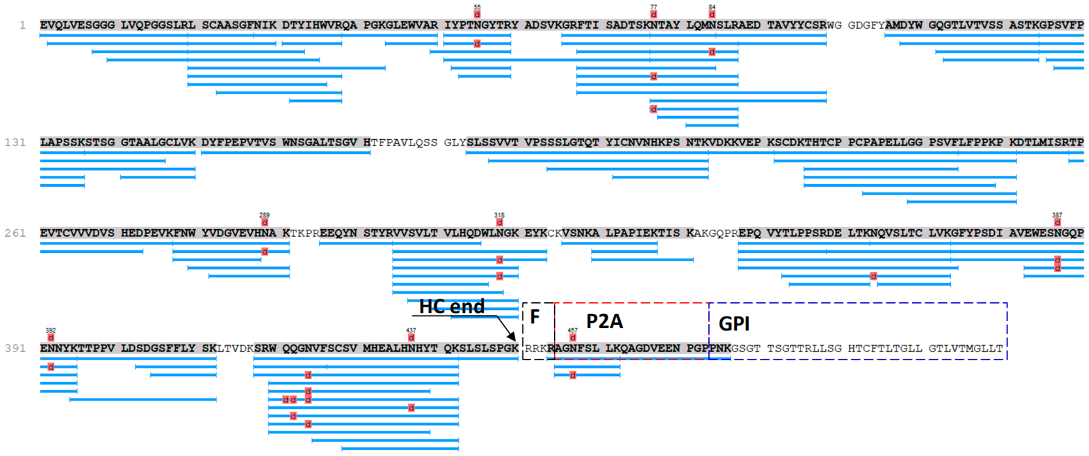

		Peptide	Unique	-10lgP	Mass	Length	ppm	m/z	RT	Area P2AT2G-up-1	Scan	#Feature	Start	End
1	☑	K.QAGDVEENPGPPNK.G	•	102.87	1450.6688	14	0.4	726.3420	20.25	8.89E5	1980	1	463	476
2		K.QAGDVEENPG.P	•	27.12	1014.4254	10	0.8	508.2204	17.46	3.68E4	1634		463	472
3		K.QAGDVEENPGPPN.K	•	16.03	1322.5739	13	-0.3	662.2940	25.28	3.32E4	2645		463	475
4	☑	R.AGNFSLLK.Q	•	53.30	848.4756	8	-0.5	425.2449	46.32	5.61E6	5229	1	455	462
5	☑	R.AGN(+.98)FSLLK.Q	•	50.37	849.4596	8	-0.3	425.7369	53.73	5.42E4	6189	1	455	462
6		R.AGN(+.98)FSLLKQ.A	•	7.18	977.5182	9	-1.1	489.7658	34.88	2.2E5	3798		455	463
7	☑	K.RAGNFSLLK.Q	•	47.92	1004.5767	9	0.7	503.2960	38.75	8.02E5	4281	1	454	462
8		K.RAGNFSLL.K	•	31.04	876.4817	8	0.0	439.2481	54.42	3.45E5	6267		454	461
9		K.RAGNFSL.L	•	26.66	763.3976	7	-0.4	382.7060	38.55	8.34E5	4248		454	460
10	☐	L.SLSPGKR.R	•	42.76	743.4290	7	-0.4	372.7216	25.08	0	2616	0	445	451
11		S.LSLSPGK.R	•	39.16	700.4119	7	0.1	351.2133	29.04	1.81E5	3086		444	450
12		S.LSLSPGKR.R	•	34.69	856.5130	8	-0.2	429.2637	25.14	0	2622		444	451
13	☑	K.SLSLSPGK.R	•	61.53	787.4440	8	-0.2	394.7292	29.04	7.54E7	3198	2	443	450

Bottom band of HC polypeptide expressed from targeting vector RRKR-P2A(T2G)

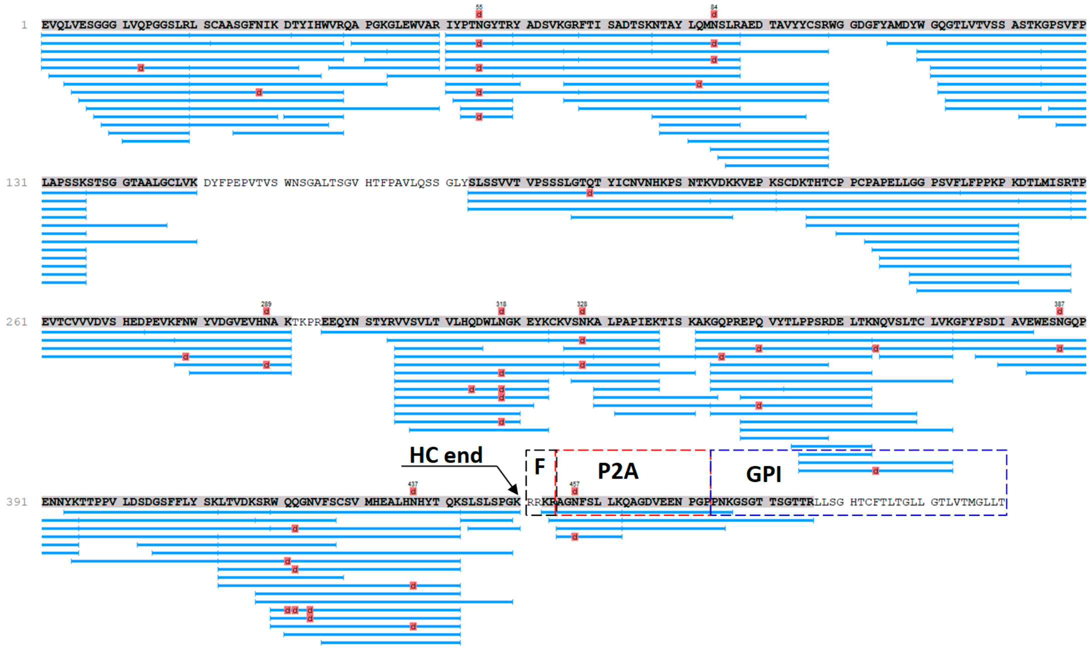

	Peptide	Unique	-10lgP	Mass	Length	ppm	m/z	RT	Area P2AT2G	Scan	#Feature	Start	End
	E.EN(+.98)PGPPN(+.98)K.G	•	5.66	853.3817	8	0.5	854.3894	54.21	5.09E5	5647		469	476
☑	K.QAGDVEENPGPPNKGSGTTSGTTR.L	•	54.52	2356.0891	24	0.1	786.3704	20.21	1.18E5	1696	1	463	486
☑	K.QAGDVEENPGPPN.K	•	45.08	1322.5739	13	0.7	662.2947	25.16	1.65E5	2164	1	463	475
☑	K.QAGDVEENPGPPNK.G	•	40.93	1450.6688	14	-0.4	726.3414	20.00	3.25E5	1655	1	463	476
	K.QAGDVEENPGPPNKGSG.T	•	9.13	1651.7438	17	-0.6	826.8787	19.95	1.52E4	1685		463	479
	K.QAGDVEENPGPPNKGSGTTS.G	•	8.06	1940.8711	20	0.2	647.9644	21.42	2.11E4	1823		463	482
	K.QAGDVEENPGPPNKGSGTTSG.T	•	5.38	1997.8926	21	0.7	666.9719	21.69	3.77E4	1840		463	483
☑	R.AGNFSLLK.Q	•	59.81	848.4756	8	0.5	425.2453	47.80	2.92E6	4824	1	455	462
☑	R.AGN(+.98)FSLLK.Q	•	50.91	849.4596	8	-0.2	425.7370	53.72	1.7E4	5602	1	455	462
	R.AGNFSLLKQAGDVEENPG.P	•	10.97	1844.8904	18	0.6	923.4531	54.54	4.72E3	5708		455	472
☑	K.RAGNFSLLK.Q	•	42.59	1004.5767	9	0.5	503.2958	39.06	1.28E6	3730	1	454	462
	K.RAGNFSL.L	•	22.99	763.3976	7	0.0	382.7061	38.71	1.73E5	3704		454	460
	K.RAGNFSLL.K	•	16.29	876.4817	8	0.3	439.2483	54.76	2.5E4	5729		454	461
☑	R.KRAGNFSLLK.Q	•	44.13	1132.6716	10	-0.3	378.5644	32.12	5.74E4	2964	1	453	462
	R.KRAGNFSLL.K	•	13.32	1004.5767	9	0.1	503.2957	45.77	1.19E4	4567		453	461
☑	S.LSLSPGK.R	•	40.95	700.4119	7	-0.5	351.2131	29.38	8.38E4	2656	1	444	450
☑	K.SLSLSPGK.R	•	59.61	787.4440	8	1.0	394.7296	29.38	3.39E7	2643	3	443	450
☑	K.SLSLSPG.K	•	52.42	659.3490	7	-0.1	330.6817	38.56	4.05E6	3680	1	443	449
	[illegible]	[illegible]	[illegible]	[illegible]	[illegible]	[illegible]	[illegible]	[illegible]	[illegible]	[illegible]		[illegible]	[illegible]

HC polypeptide expressed from targeting vector RRKR-P2A(A1P)

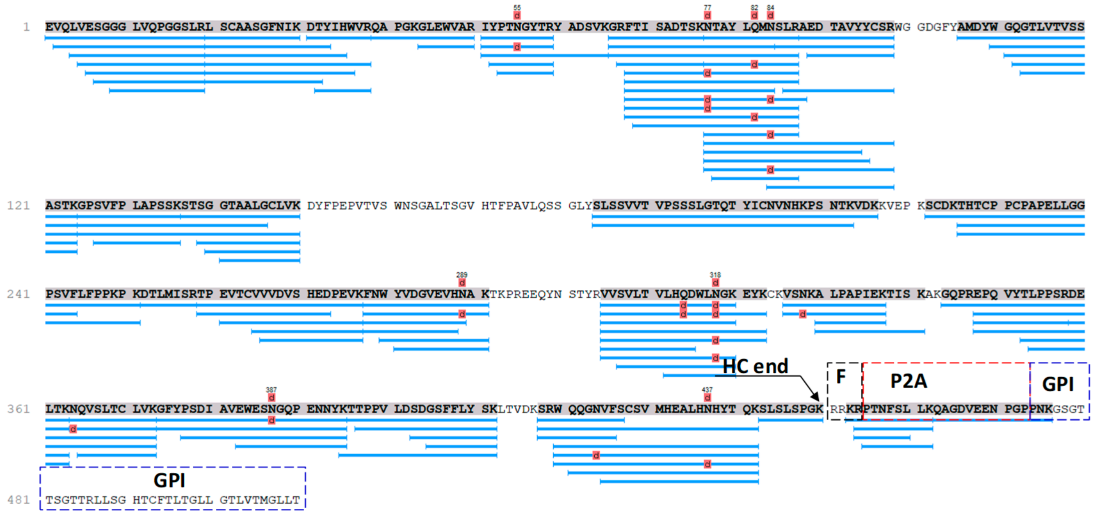

☑ K.QAGDVEENPGPPNK.G	•	67.86	1450.6688	14	0.7	726.3422	19.84	1.04E6	1656	1	463	476
K.QAGDVEENPGPPN.K	•	13.28	1322.5739	13	0.1	662.2943	25.08	3.05E5	2139		463	475
K.QAGDVEENPGPPNKGSGTTSGTTR.L	•	9.20	2356.0891	24	1.0	786.3711	20.06	4.94E4	1703		463	486
☑ R.PTNFSLLK.Q	•	51.74	918.5175	8	-0.2	460.2659	49.09	1.46E4	5002	1	455	462
☑ K.RPTNFSLLK.Q	•	58.64	1074.6185	9	-1.1	359.2131	41.34	9.22E5	4030	2	454	462
☐ K.RPTNFSLL.K	•	44.19	946.5236	8	-0.3	474.2689	55.79	0	5866	0	454	461
☑ K.RPTNFSL.L	•	42.87	833.4395	7	-0.1	417.7270	42.27	5.44E6	4087	1	454	460
☑ R.KRPTNFSLLK.Q	•	52.05	1202.7135	10	-1.5	401.9112	34.83	8.95E5	3266	2	453	462

HC polypeptide expressed from targeting vector RRKR-P2A(T2P)

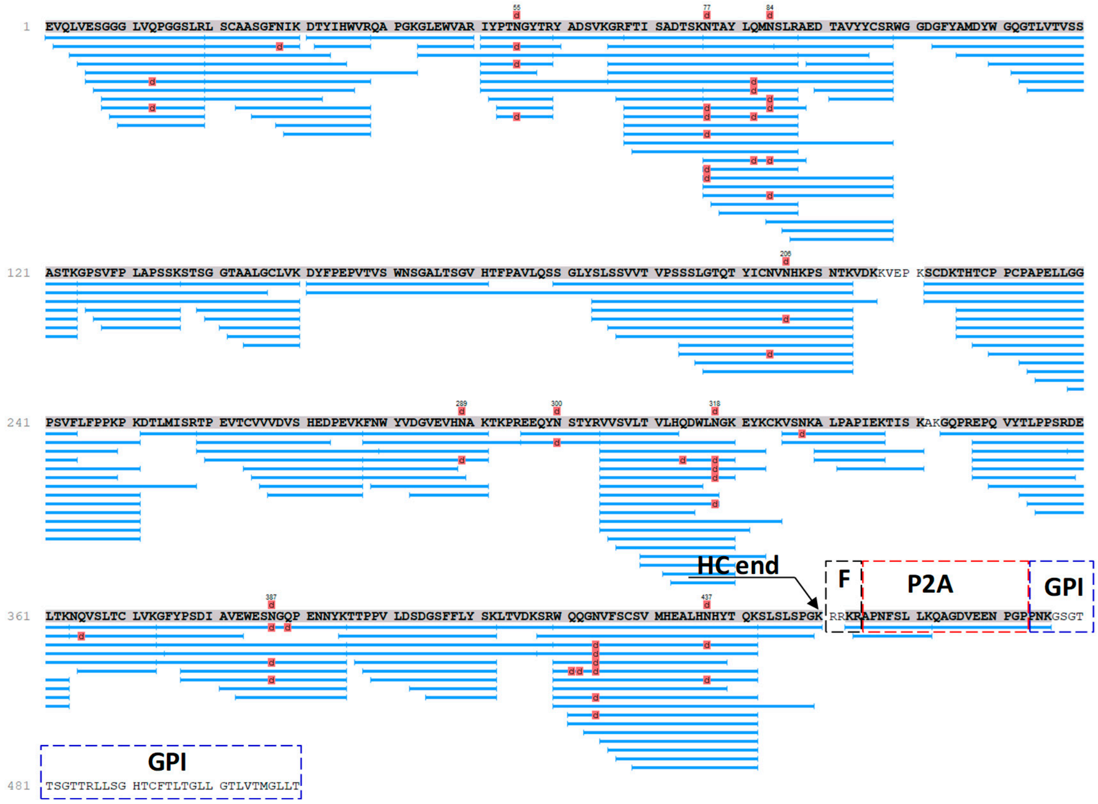

☑	K.QAGDVEENPGPPNK.G	•	102.42	1450.6688	14	0.0	726.3417	20.02	3.72E5	1736	1	463	476
	K.QAGDVEENPGPPN.K	•	14.07	1322.5739	13	0.8	662.2947	25.11	3.22E4	2275		463	475
☑	K.RAPNFSLLK.Q	•	44.28	1044.6079	9	0.3	523.3114	44.75	1.86E5	4647	1	454	462
	K.RAPNFSLL.K	•	24.76	916.5130	8	-0.6	459.2635	59.06	1.15E4	6531		454	461
	K.RAPN(+.98)FSLLKQ(+.98).A	•	6.48	1174.6345	10	3.9	588.3268	51.36	0	5529		454	463
☑	R.KRAPNFSLLK.Q	•	45.03	1172.7029	10	-0.5	391.9081	37.03	5.43E4	3715	1	453	462
	R.KRAPNFSL.L	•	19.19	931.5239	8	0.7	466.7696	36.35	2.35E4	3618		453	460
☑	K.SLSLSPGK.R	•	62.74	787.4440	8	0.0	394.7292	29.14	1.32E7	2760	2	443	450
☑	K.SLSLSPG.K	•	53.15	659.3490	7	-0.7	330.6815	38.39	1.42E6	3858	1	443	449

HC polypeptide expressed from targeting vector RRKR-P2A(N3P)

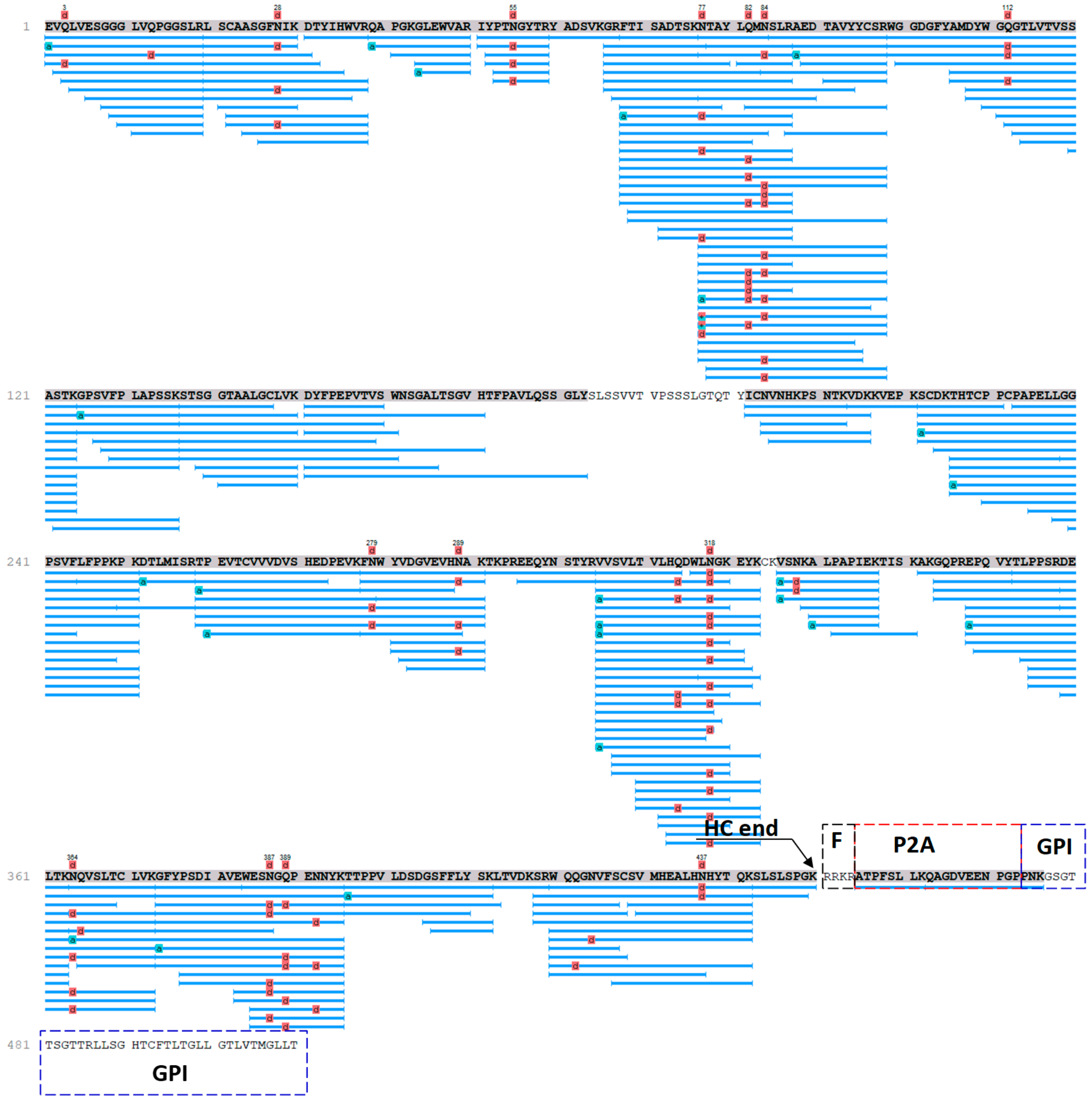

☑	K.QAGDVEENPGPPNK.G	•	81.64	1450.6...	14	0.7	726.3...	20...	1.85E4	3283	1	463	476
	K.Q(+.98)AGDVEENPGPPNKGSGT...	•	15.59	2357.0...	24	7.1	786.7...	51...	0	9251		463	486
	K.QAGDVEENPGPPNKGSGTTSGTTR.L	•	9.51	2356.0...	24	-3.7	786.3...	79...	5.5E4	14834		463	486
	K.QAGDVEENPGPP.N	•	6.97	1208.5...	12	5.9	403.8...	54...	2.77E5	10001		463	474
	F.S(+42.01)LLKQ(+.98)AGDVEEN...	•	8.40	1935.9...	18	-2.0	646.3...	45...	1.76E6	8180		459	476
☑	R.ATPFSLLK.Q	•	52.04	875.5...	8	-0.3	438.7...	63...	1.18E4	11614	1	455	462
	R.ATPFSLLKQ(+.98)AGDVEENPGP...	•	8.76	2310.1...	22	-7.4	578.5...	63...	2.36E5	11657		455	476
	R.ATPFSLLKQAGDVEEN(+.98)PGP...	•	7.90	2310.1...	22	-8.3	578.5...	61...	0	11321		455	476
	R.ATPFSLLKQ(+.98)AGDVEEN(+....	•	6.59	2311.1...	22	9.0	1156.5...	74...	0	13870		455	476
	R.ATPFSLLKQ(+.98)AGDVEENPGP...	•	5.71	2309.1...	22	-6.2	770.7...	39...	0	6765		455	476
	K.R(+42.01)ATPFSLLKQAGDVEEN...	•	8.19	2506.2...	23	6.1	836.4...	67...	2.01E4	12482		454	476
☑	K.SLSLSPGK.R	•	57.56	787.4...	8	1.0	394.7...	32...	4.17E6	5571	1	443	450
☑	K.SLSLSPG.K	•	46.66	659.3...	7	0.0	330.6...	41...	2.05E5	7191	1	443	449

HC polypeptide expressed from targeting vector RRKR-P2A(F4P)

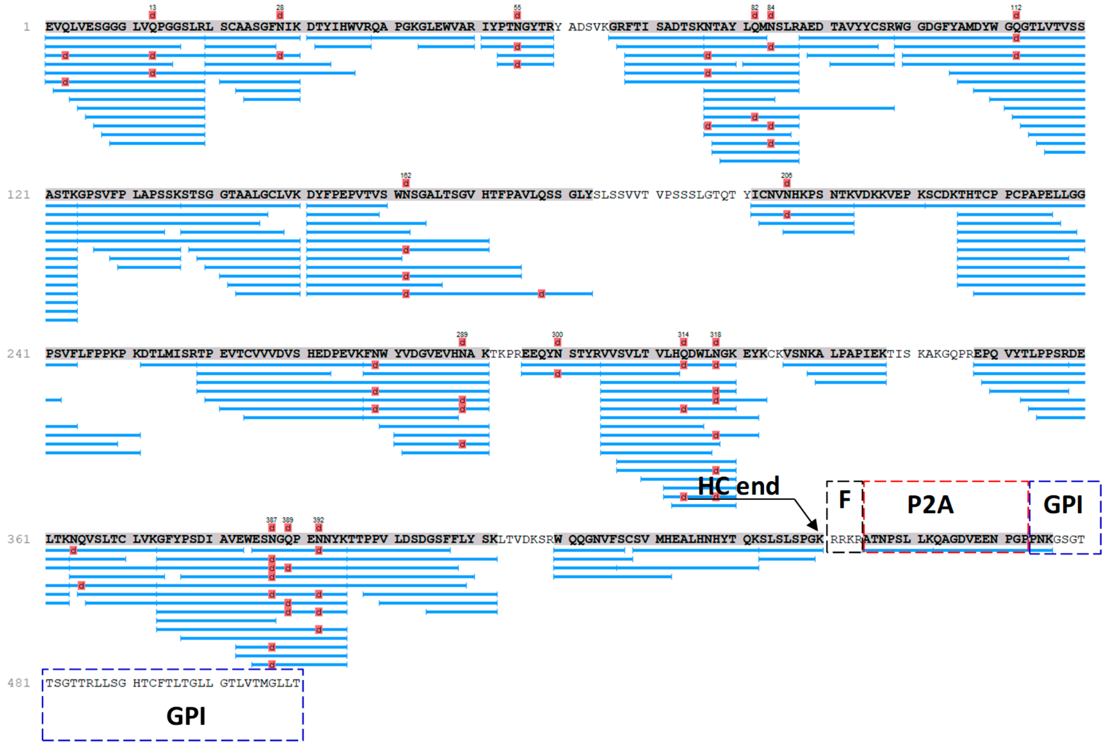

1		V.EENPGPPNKGSGTTSGTTR.L	•	8.47	1885.8...	19	5.6	943.9...	75...	2.31E5	14223		468	486
2	☑	K.QAGDVEENPGPPNK.G	•	87.91	1450.6...	14	0.0	726.3...	20...	1.23E5	3296	1	463	476
3	☐	K.QAGDVEENPGPPN.K	•	58.73	1322.5...	13	0.0	662.2...	24...	0	4157	0	463	475
4	☐	K.QAGDVEENPG.P	•	45.28	1014.4...	10	0.2	508.2...	18...	0	2975	0	463	472
5	☑	R.ATNPSLLK.Q	•	51.39	842.4...	8	1.4	422.2...	31...	3.46E4	5325	1	455	462
6		K.RATNPSLLKQAGDVEEN.P	•	6.76	1840.9...	17	6.9	614.6...	44...	0	7967		454	470
7		S.LSLSPGK.R	•	21.16	700.4...	7	1.3	351.2...	32...	0	5686		444	450
8	☑	K.SLSLSPGK.R	•	53.19	787.4...	8	1.4	394.7...	34...	4.35E6	5940	1	443	450
9	☑	K.SLSLSPG.K	•	46.53	659.3...	7	-0.6	330.6...	41...	1.98E5	7282	1	443	449

HC polypeptide expressed from targeting vector RRKR-P2A(S5P)

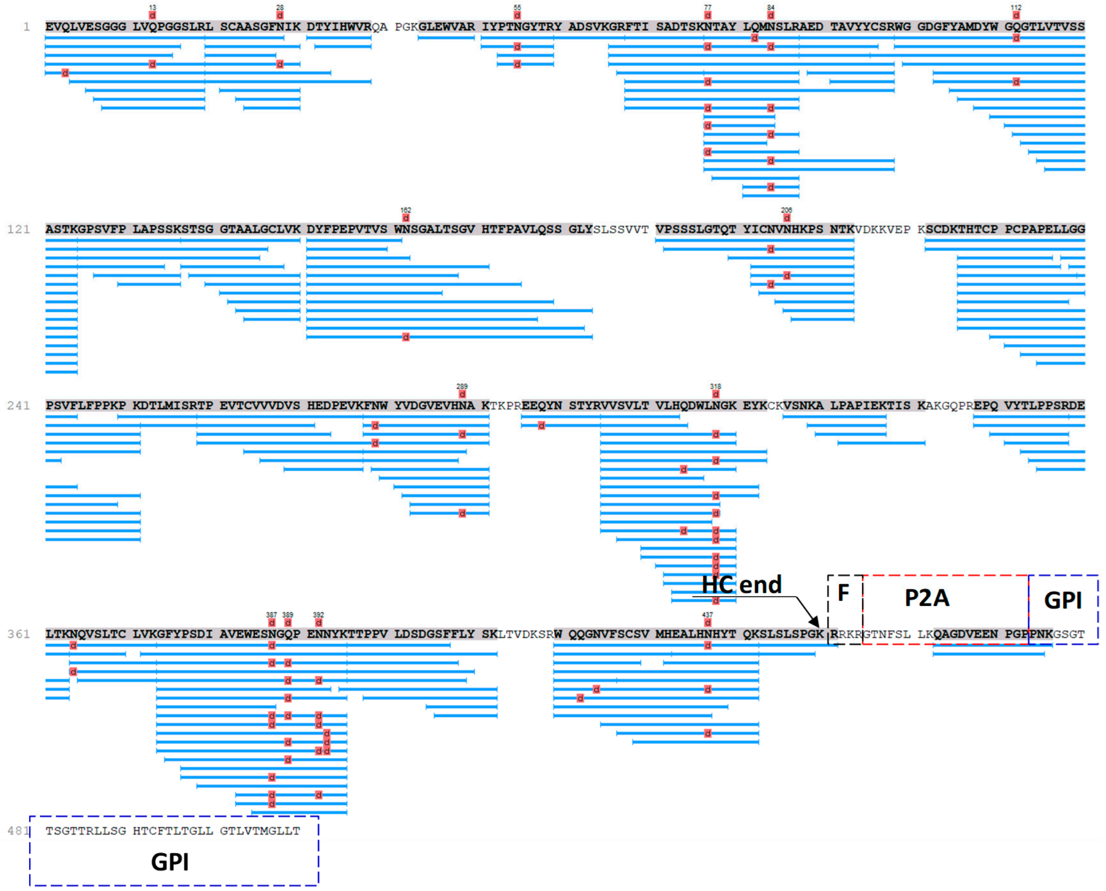

	Peptide	Unique	-10lgP	Mass	Length	ppm	m/z	RT	Area P2AS5P_GEL2	Scan	#Feature	St...	End
☑	K.QAGDVEENPGPPNK.G	•	79.62	1450.6...	14	0.5	726.3...	20...	3E5	3240	1	463	476
☑	K.QAGDVEENPGPPN.K	•	64.97	1322.5...	13	-0.4	662.2...	24...	3.85E4	4079	1	463	475
	K.Q(+.98)AGDVEEN(+.98)PGPPNK...	•	7.56	2358.0...	24	-5.6	787.0...	65...	0	12303		463	486
	K.Q(+.98)AGDVEENPGPPNK.G	•	5.35	1451.6...	14	-6.8	726.8...	76...	6.92E6	14356		463	476
	L.LKQAGDVEENPGPPNKGSGTTSGT...	•	8.48	2597.2...	26	1.7	866.7...	74...	6.44E3	14095		461	486
	G.TNFSLLKQAGDVEENPGPPNK.G	•	8.32	2254.1...	21	-4.1	752.3...	71...	0	13554		456	476
	R.GTN(+.98)FSLLKQ(+.98)AGDVE...	•	5.86	3219.5...	32	4.3	644.9...	61...	0	11314		455	486
	K.RGTNFSLLKQAGDVEEN(+.98)PG...	•	21.08	3373.6...	33	2.1	675.7...	74...	0	13973		454	486
	K.RGTNFSLLKQAGDVEEN(+.98)PG...	•	20.82	3374.6...	33	9.5	675.9...	73...	3.38E4	13973		454	486
	R.KRGTN(+.98)FSLLKQ(+.98)AGD...	•	5.64	2598.2...	24	1.0	867.1...	47...	0	8544		453	476
☑	K.SLSLSPGKR.R	•	54.71	943.5...	9	-0.4	472.7...	27...	2.2E4	4556	1	443	451
☑	K.SLSLSPG.K	•	39.33	659.3...	7	1.2	330.6...	41...	9.46E5	7226	2	443	449

HC polypeptide expressed from targeting vector RRKR-P2A(N3A)

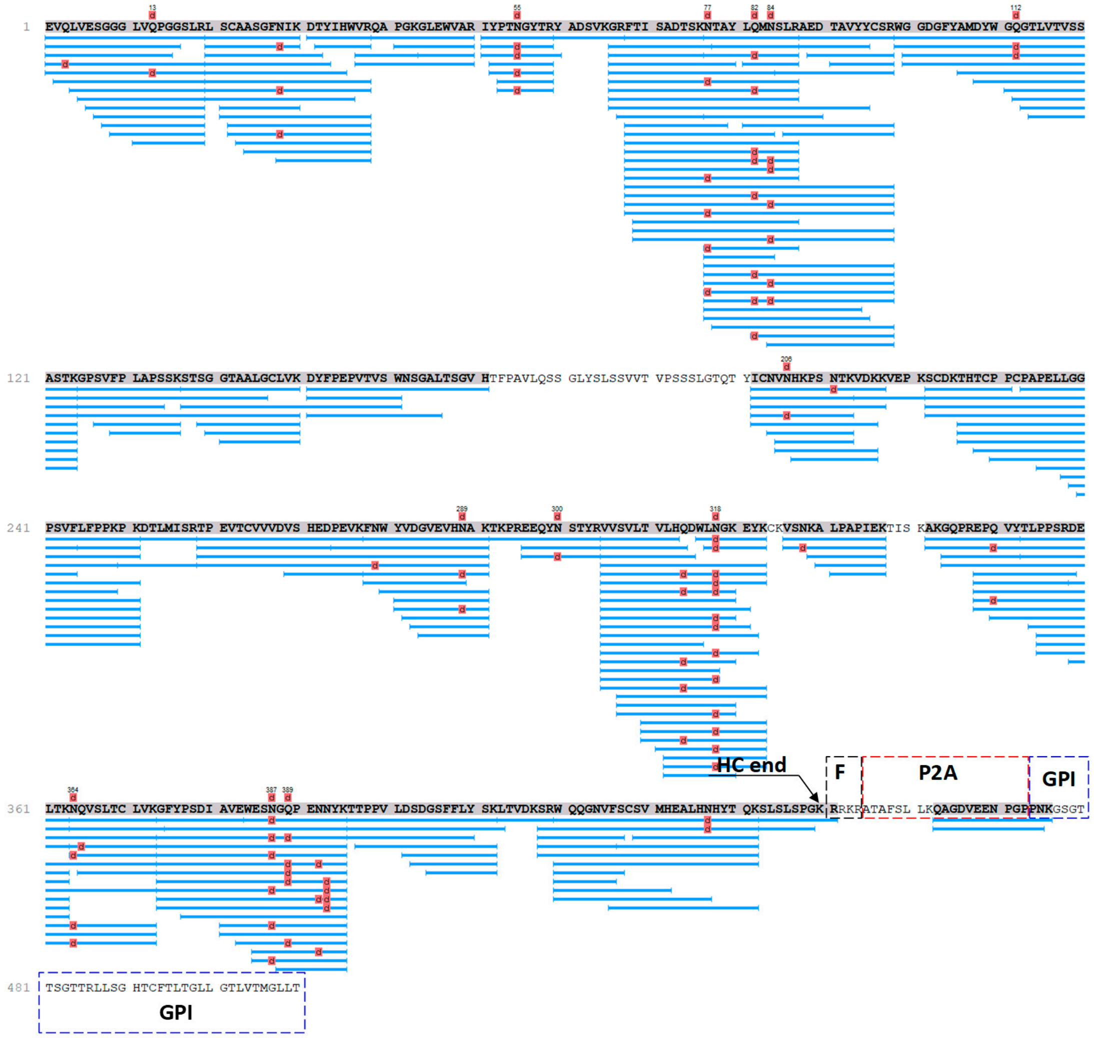

	G.DVEENPGPPNK.G	•	8.31	1194.5516	11	-1.2	598.2823	66.48	1.94E4	12021		466	476
☑	K.QAGDVEENPGPPNK.G	•	80.85	1450.6688	14	-0.9	726.3410	20.17	9.11E3	3165	1	463	476
☑	K.QAGDVEENPGPPN.K	•	59.17	1322.5739	13	-0.2	662.2941	24.62	0	3927	0	463	475
	R.ATAFSLLKQ(+.98)AGDVEENPGPPNK.G	•	7.18	2283.1382	22	9.0	762.0602	74.57	3.73E4	13650		455	476
☑	K.SLSLSPG.K	•	46.06	659.3490	7	0.0	330.6818	41.47	8.11E5	6939	2	443	449
☑	K.SLSLSPGKR.R	•	29.18	943.5450	9	-0.8	315.5220	27.53	1.67E5	4430	1	443	451

HC polypeptide expressed from targeting vector RRKR-P2A(F4A)

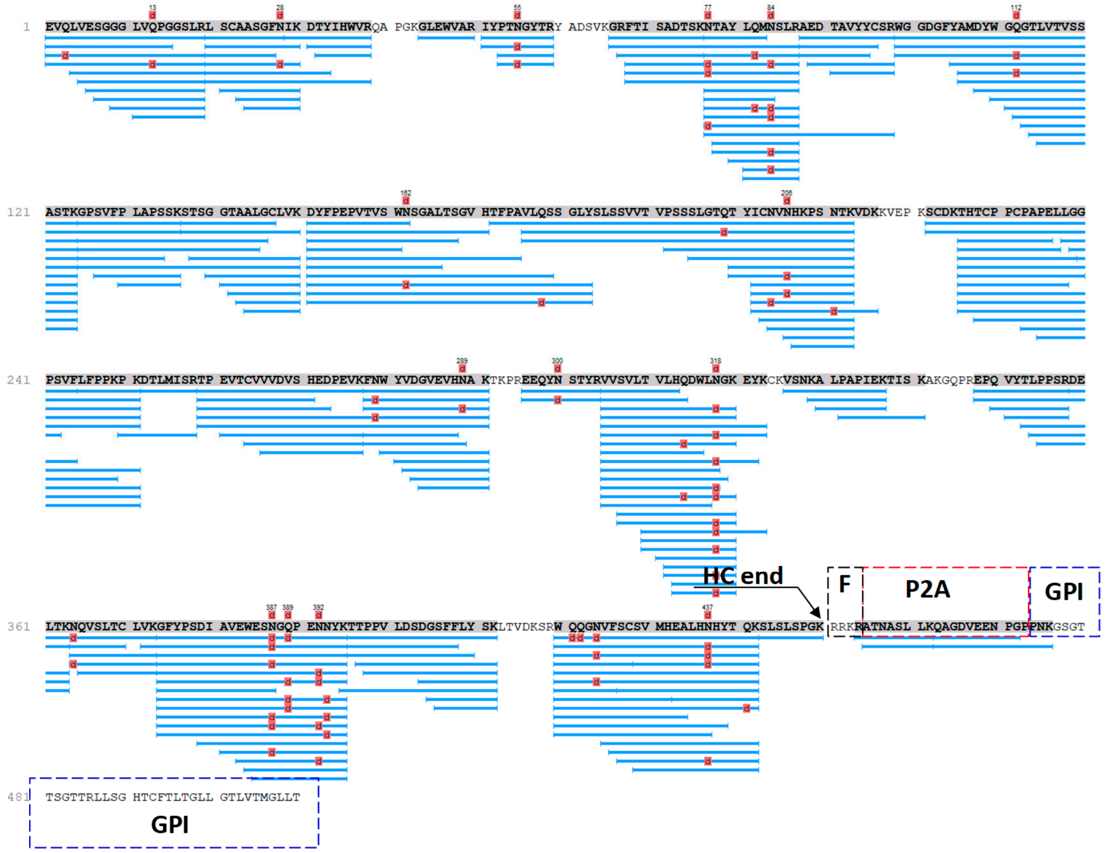

1	☑	K.QAGDVEENPGPPNK.G	•	94.93	1450.6688	14	-0.2	726.3416	20.02	6.6E4	3178	2	463	476
2	☑	K.QAGDVEENPG.P	•	57.79	1014.4254	10	-0.6	508.2197	18.36	3.24E4	2867	1	463	472
3	☑	K.QAGDVEENPGPPN.K	•	52.97	1322.5739	13	-0.2	662.2941	24.58	0	3999	0	463	475
4		K.Q(+.98)AGDVEENPGPPNK.G	•	5.66	1451.6528	14	-7.3	726.8284	76.01	6.03E6	14517		463	476
5		T.N(+.98)ASLLKQAGDVEENPGPPN(+....	•	18.46	2079.0120	20	-0.8	694.0107	56.69	6.02E4	10273		457	476
6	☑	R.ATNASLLK.Q	•	70.82	816.4705	8	0.3	409.2426	28.67	6.53E4	4729	1	455	462
7		R.ATN(+.98)ASLLK.Q	•	27.03	817.4545	8	-0.3	409.7344	31.84	0	5338		455	462
8	☑	K.RATNASLLK.Q	•	52.97	972.5716	9	0.9	487.2935	23.19	8.64E3	3741	1	454	462
9	☑	K.SLSLSPGK.R	•	55.52	787.4440	8	-0.8	394.7289	32.62	5.15E6	5432	1	443	450

HC polypeptide expressed from targeting vector RRKRGTNFS

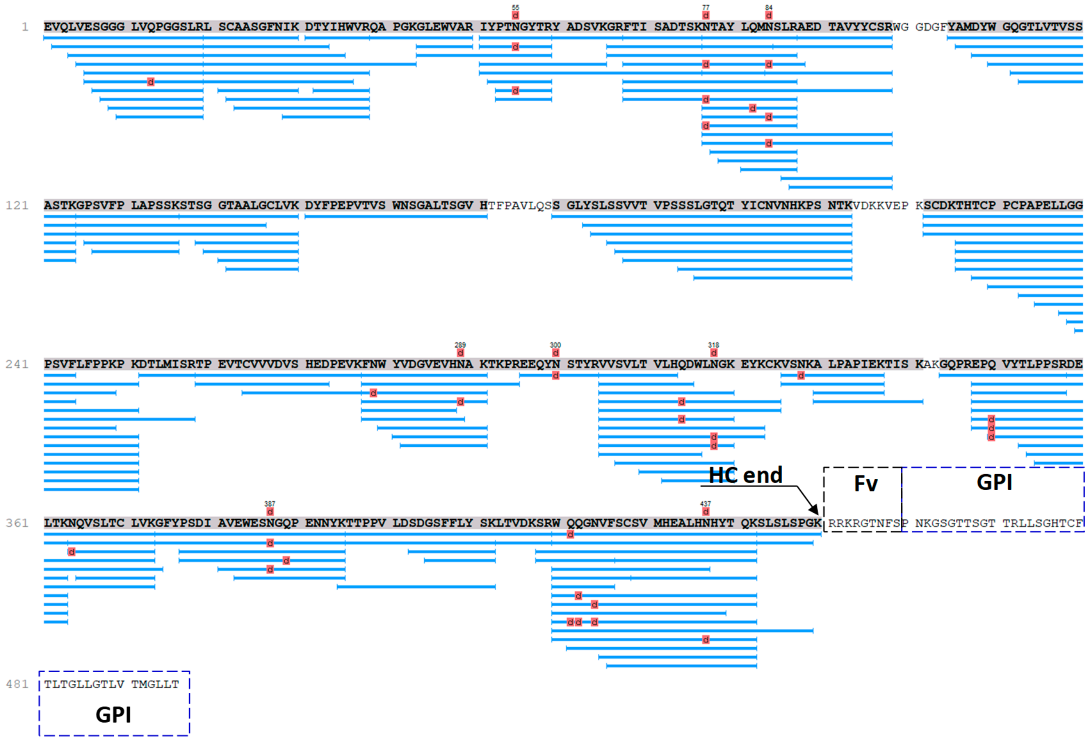

Top band of HC polypeptide expressed from targeting vector RRKRAGNFS

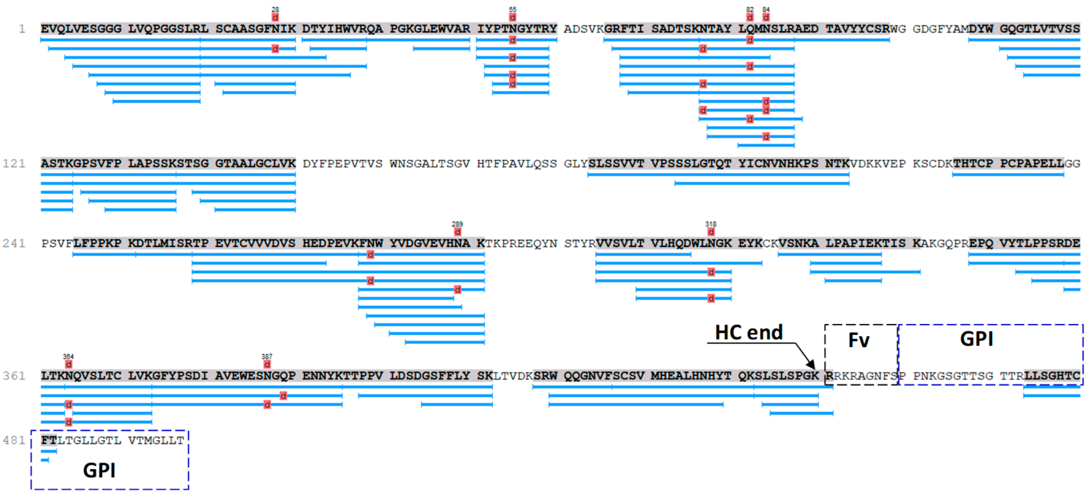

1	☑	R.LLSGHTC(+57.02)FT.L	•	51.51	1034.4855	9	0.2	518.2501	37.18	2.1E4	3561	1	474	482
2	☑	R.LLSGHTC(+57.02)F.T	•	47.51	933.4378	8	0.2	467.7263	38.61	2.13E3	3726	1	474	481
3		P.NKGSGTTSGTTR.L	•	6.98	1165.5687	12	-5.2	583.7886	30.55	4.03E3	2836		462	473
4	☑	L.SLSPGKR.R	•	48.97	743.4290	7	0.4	372.7219	24.37	2.01E5	2140	1	445	451
5	☑	S.LSLSPGK.R	•	40.19	700.4119	7	0.7	351.2135	28.36	8.27E4	2575	1	444	450
6		S.LSLSPGKR.R	•	16.36	856.5130	8	-0.1	429.2637	24.37	3.43E4	2156		444	451
7	☑	K.SLSLSPGK.R	•	59.84	787.4440	8	0.0	394.7292	28.36	3.21E7	2556	2	443	450
8	☑	K.SLSLSPGKR.R	•	37.62	943.5450	9	-0.7	472.7794	24.37	2.84E5	2126	1	443	451

Bottom band of HC polypeptide expressed from targeting vector RRKRAGNFS

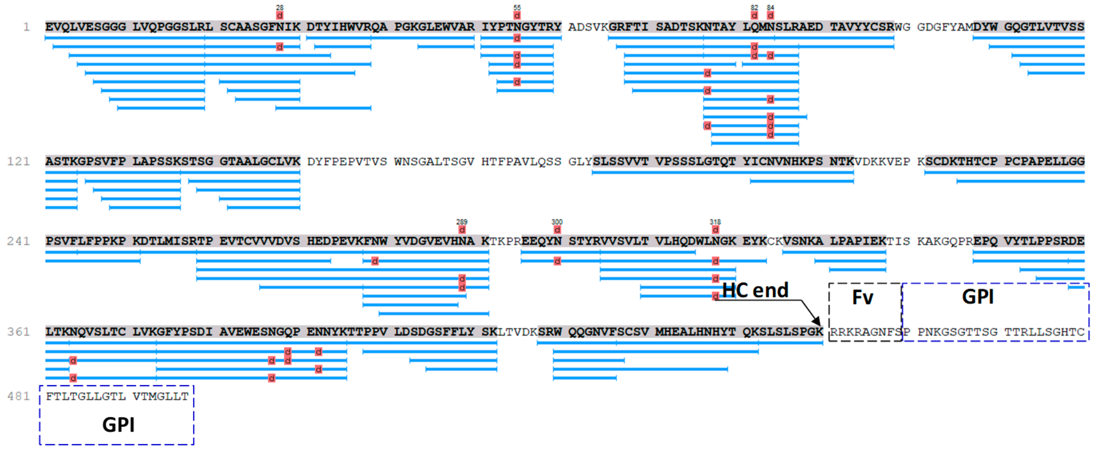

1	☐	L.SLSPGKR.R	•	25.83	743.4290	7	0.0	372.7218	24.18	0	2140	0	445	451
2	☐	S.LSLSPGK.R	•	38.76	700.4119	7	0.8	351.2135	28.37	0	2581	0	444	450
3	☑	K.SLSLSPGK.R	•	54.05	787.4440	8	-0.6	394.7290	28.15	3.62E5	2557	2	443	450
4	☐	K.SLSLSPGKR.R	•	33.38	943.5450	9	-0.1	472.7798	24.10	0	2133	0	443	451
5	☑	F.SC(+57.02)SVM(+15.99)HEALHNHY…	•	26.33	1956.8571	16	0.3	490.2217	19.54	1.69E5	1736	1	427	442
6	☑	R.WQQGNVFSC(+57.02)SVM(+15.99…	•	91.01	2816.2546	23	0.0	705.0709	43.13	4.95E5	4185	1	420	442

Supplementary Figure S2

Multiple alignment of 1:1:1 Top% sorted 10 clones VH

```
CLUSTAL O(1.2.4) multiple sequence alignment


Herceptin-VH      EVQLVESGGGLVQPGGSLRLSCAASGFNIKDTYIHWVRQAPGKGLEWVARIYPTNGYTRY      60
CL1-HC-1          EVQLVESGGGLVQPGGSLRLSCAASGFNIKDTYIHWVRQAPGKGLEWVARIYPTNGYTRY      60
CL2-HC-2          EVQLVESGGGLVQPGGSLRLSCAASGFNIKDTYIHWVRQAPGKGLEWVARIYPTNGYTRY      60
CL3-HC-3          EVQLVESGGGLVQPGGSLRLSCAASGFNIKDTYIHWVRQAPGKGLEWVARIYPTNGYTRY      60
CL4-HC-4          EVQLVESGGGLVQPGGSLRLSCAASGFNIKDTYIHWVRQAPGKGLEWVARIYPTNGYTRY      60
CL5-HC-5          EVQLVESGGGLVQPGGSLRLSCAASGFNIKDTYIHWVRQAPGKGLEWVARIYPTNGYTRY      60
CL6-HC-6          EVQLVESGGGLVQPGGSLRLSCAASGFNIKDTYIHWVRQAPGKGLEWVARIYPTNGYTRY      60
CL7-HC-7          EVQLVESGGGLVQPGGSLRLSCAASGFNIKDTYIHWVRQAPGKGLEWVARIYPTNGYTRY      60
CL8-HC-8          EVQLVESGGGLVQPGGSLRLSCAASGFNIKDTYIHWVRQAPGKGLEWVARIYPTNGYTRY      60
CL9-HC-9          EVQLVESGGGLVQPGGSLRLSCAASGFNIKDTYIHWVRQAPGKGLEWVARIYPTNGYTRY      60
CL10-HC-10        EVQLVESGGGLVQPGGSLRLSCAASGFNIKDTYIHWVRQAPGKGLEWVARIYPTNGYTRY      60
                  ************************************************************

Herceptin-VH      ADSVKGRFTISADTSKNTAYLQMNSLRAEDTAVYYCSRWGGDGFYAMDYWGQGTLVTVSS      120
CL1-HC-1          ADSVKGRFTISADTSKNTAYLQMNSLRAEDTAVYYCSRWGGDGFYAMDYWGQGTLVTVSS      120
CL2-HC-2          ADSVKGRFTISADTSKNTAYLQMNSLRAEDTAVYYCSRWGGDGFYAMDYWGQGTLVTVSS      119
CL3-HC-3          ADSVKGRFTISADTSKNTAYLQMNSLRAEDTAVYYCSRWGGDGFYAMDYWGQGTLVTVSS      119
CL4-HC-4          ADSVKGRFTISADTSKNTAYLQMNSLRAEDTAVYYCSRWGGDGFYAMDYWGQGTLVTVSS      119
CL5-HC-5          ADSVKGRFTISADTSKNTAYLQMNSLRAEDTAVYYCSRWGGDGFYAMDYWGQGTLVTVSS      119
CL6-HC-6          ADSVKGRFTISADTSKNTAYLQMNSLRAEDTAVYYCSRWGGDGFYAMDYWGQGTLVTVSS      119
CL7-HC-7          ADSVKGRFTISADTSKNTAYLQMNSLRAEDTAVYYCSRWGGDGFYAMDYWGQGTLVTVSS      119
CL8-HC-8          ADSVKGRFTISADTSKNTAYLQMNSLRAEDTAVYYCSRWGGDGFYAMDYWGQGTLVTVSS      119
CL9-HC-9          ADSVKGRFTISADTSKNTAYLQMNSLRAEDTAVYYCSRWGGDGFYAMDYWGQGTLVTVSS      119
CL10-HC-10        ADSVKGRFTISADTSKNTAYLQMNSLRAEDTAVYYCSRWGGDGFYAMDYWGQGTLVTVSS      119
                  ************************************************************
```

Multiple alignment of 1:1:1 Top% sorted 10 clones VL

```
CLUSTAL O(1.2.4) multiple sequence alignment


Herceptin-VL      DIQMTQSPSSLSASVGDRVTITCRASQDVNTAVAWYQQKPGKAPKLLIYSASFLYSEVPS      60
CL1-LC-1          DIQMTQSPSSLSASVGDRVTITCRASQDVNTAVAWYQQKPGKAPKLLIYSASFLYSEVPS      60
CL2-LC-2          DIQMTQSPSSLSASVGDRVTITCRASQDVNTAVAWYQQKPGKAPKLLIYSASFLYSEVPS      60
CL3-LC-3          DIQMTQSPSSLSASVGDRVTITCRASQDVNTAVAWYQQKPGKAPKLLIYSASFLYSEVPS      60
CL4-LC-4          DIQMTQSPSSLSASVGDRVTITCRASQDVNTAVAWYQQKPGKAPKLLIYSASFLYSEVPS      60
CL5-LC-5          DIQMTQSPSSLSASVGDRVTITCRASQDVNTAVAWYQQKPGKAPKLLIYSASFLYSEVPS      60
CL6-LC-6          DIQMTQSPSSLSASVGDRVTITCRASQDVNTAVAWYQQKPGKAPKLLIYSASFLYSEVPS      60
CL7-LC-7          DIQMTQSPSSLSASVGDRVTITCRASQDVNTAVAWYQQKPGKAPKLLIYSASFLYSEVPS      60
CL8-LC-8          DIQMTQSPSSLSASVGDRVTITCRASQDVNTAVAWYQQKPGKAPKLLIYSASFLYSEVPS      60
CL9-LC-9          DIQMTQSPSSLSASVGDRVTITCRASQDVNTAVAWYQQKPGKAPKLLIYSASFLYSEVPS      60
CL10-LC-10        DIQMTQSPSSLSASVGDRVTITCRASQDVNTAVAWYQQKPGKAPKLLIYSASFLYSEVPS      60
                  ************************************************************

Herceptin-VL      RFSGSRSGTDFTLTISSLQPEDFATYYCQQHYTTPPTFGQGTKVEIK      107
CL1-LC-1          RFSGSRSGTDFTLTISSLQPEDFATYYCQQHYTTPPTFGQGTKVEIK      107
CL2-LC-2          RFSGSRSGTDFTLTISSLQPEDFATYYCQQHYTTPPTFGQGTKVEIK      107
CL3-LC-3          RFSGSRSGTDFTLTISSLQPEDFATYYCQQHYTTPPTFGQGTKVEIK      107
CL4-LC-4          RFSGSRSGTDFTLTISSLQPEDFATYYCQQHYTTPPTFGQGTKVEIK      107
CL5-LC-5          RFSGSRSGTDFTLTISSLQPEDFATYYCQQHYTTPPTFGQGTKVEIK      107
CL6-LC-6          RFSGSRSGTDFTLTISSLQPEDFATYYCQQHYTTPPTFGQGTKVEIK      107
CL7-LC-7          RFSGSRSGTDFTLTISSLQPEDFATYYCQQHYTTPPTFGQGTKVEIK      107
CL8-LC-8          RFSGSRSGTDFTLTISSLQPEDFATYYCQQHYTTPPTFGQGTKVEIK      107
CL9-LC-9          RFSGSRSGTDFTLTISSLQPEDFATYYCQQHYTTPPTFGQGTKVEIK      107
CL10-LC-10        RFSGSRSGTDFTLTISSLQPEDFATYYCQQHYTTPPTFGQGTKVEIK      107
                  ***********************************************
```

Multiple alignment of 10 VH 0.01% Sorted clones

```
CLUSTAL O (1.2.4) multiple sequence alignment


Herceptin_VH      EVQLVESGGGLVQPGGSLRLSCAASGFNIKDTYIHWVRQAPGKGLEWVARIYPTNGYTRY      60
C1_VH_0-01        EVQLVESGGGLVQPGGSLRLSCAASGFNIKDTYIHWVRQAPGKGLEWVARIYPTNGYTRY      60
C2_VH_0-01        EVQLVESGGGLVQPGGSLRLSCAASGFNIKDTYIHWVRQAPGKGLEWVARIYPTNGYTRY      60
C3_VH_0-01        EVQLVESGGGLVQPGGSLRLSCAASGFNIKDTYIHWVRQAPGKGLEWVARIYPTNGYTRY      60
C4_VH_0-01        EVQLVESGGGLVQPGGSLRLSCAASGFNIKDTYIHWVRQAPGKGLEWVARIYPTNGYTRY      60
C5_VH_0-01        EVQLVESGGGLVQPGGSLRLSCAASGFNIKDTYIHWVRQAPGKGLEWVARIYPTNGYTRY      60
C6_VH_0-01        EVQLVESGGGLVQPGGSLRLSCAASGFNIKDTYIHWVRQAPGKGLEWVARIYPTNGYTRY      60
C7_VH_0-01        EVQLVESGGGLVQPGGSLRLSCAASGFNIKDTYIHWVRQAPGKGLEWVARIYPTNGYTRY      60
C8_VH_0-01        EVQLVESGGGLVQPGGSLRLSCAASGFNIKDTYIHWVRQAPGKGLEWVARIYPTNGYTRY      60
C9_VH_0-01        EVQLVESGGGLVQPGGSLRLSCAASGFNIKDTYIHWVRQAPGKGLEWVARIYPTNGYTRY      60
C10_VH_0-01       EVQLVESGGGLVQPGGSLRLSCAASGFNIKDTYIHWVRQAPGKGLEWVARIYPTNGYTRY      60
                  ************************************************************

Herceptin_VH      ADSVKGRFTISADTSKNTAYLQMNSLRAEDTAVYYCSRWGGDGFYAMDYWGQGTLVTVSS      120
C1_VH_0-01        ADSVKGRFTISADTSKNTAYLQMNSLRAEDTAVYYCSRWGGDGFYAMDYWGQGTLVTVSS      120
C2_VH_0-01        ADSVKGRFTISADTSKNTAYLQMNSLRAEDTAVYYCSRWGGDGFYAMDYWGQGTLVTVSS      120
C3_VH_0-01        ADSVKGRFTISADTSKNTAYLQMNSLRAEDTAVYYCSRWGGDGFYAMDYWGQGTLVTVSS      120
C4_VH_0-01        ADSVKGRFTISADTSKNTAYLQMNSLRAEDTAVYYCSRWGGDGFYAMDYWGQGTLVTVSS      120
C5_VH_0-01        ADSVKGRFTISADTSKNTAYLQMNSLRAEDTAVYYCSRWGGDGFYAMDYWGQGTLVTVSS      120
C6_VH_0-01        ADSVKGRFTISADTSKNTAYLQMNSLRAEDTAVYYCSRWGGDGFYAMDYWGQGTLVTVSS      120
C7_VH_0-01        ADSVKGRFTISADTSKNTAYLQMNSLRAEDTAVYYCSRWGGDGFYAMDYWGQGTLVTVSS      120
C8_VH_0-01        ADSVKGRFTISADTSKNTAYLQMNSLRAEDTAVYYCSRWGGDGFYAMDYWGQGTLVTVSS      120
C9_VH_0-01        ADSVKGRFTISADTSKNTAYLQMNSLRAEDTAVYYCSRWGGDGFYAMDYWGQGTLVTVSS      120
C10_VH_0-01       ADSVKGRFTISADTSKNTAYLQMNSLRAEDTAVYYCSRWGGDGFYAMDYWGQGTLVTVSS      120
                  ************************************************************
```

Multiple alignment of 10 VL 0.01% Sorted clones

```
CLUSTAL O(1.2.4) multiple sequence alignment


Herceptin         DIQMTQSPSSLSASVGDRVTITCRASQDVNTAVAWYQQKPGKAPKLLIYSASFLYSEVPS      60
C1_VL_0-01        DIQMTQSPSSLSASVGDRVTITCRASQDVNTAVAWYQQKPGKAPKLLIYSASFLYSEVPS      60
C2_VL_0-01        DIQMTQSPSSLSASVGDRVTITCRASQDVNTAVAWYQQKPGKAPKLLIYSASFLYSEVPS      60
C3_VL_0-01        DIQMTQSPSSLSASVGDRVTITCRASQDVNTAVAWYQQKPGKAPKLLIYSASFLYSEVPS      60
C4_VL_0-01        DIQMTQSPSSLSASVGDRVTITCRASQDVNTAVAWYQQKPGKAPKLLIYSASFLYSEVPS      60
C5_VL_0-01        DIQMTQSPSSLSASVGDRVTITCRASQDVNTAVAWYQQKPGKAPKLLIYSASFLYSEVPS      60
C6_VL_0-01        DIQMTQSPSSLSASVGDRVTITCRASQDVNTAVAWYQQKPGKAPKLLIYSASFLYSEVPS      60
C7_VL_0-01        DIQMTQSPSSLSASVGDRVTITCRASQDVNTAVAWYQQKPGKAPKLLIYSASFLYSEVPS      60
C8_VL_0-01        DIQMTQSPSSLSASVGDRVTITCRASQDVNTAVAWYQQKPGKAPKLLIYSASFLYSEVPS      60
C9_VL_0-01        DIQMTQSPSSLSASVGDRVTITCRASQDVNTAVAWYQQKPGKAPKLLIYSASFLYSEVPS      60
C10_VL_0-01       DIQMTQSPSSLSASVGDRVTITCRASQDVNTAVAWYQQKPGKAPKLLIYSASFLYSEVPS      60
                  ************************************************************

Herceptin         RFSGSRSGTDFTLTISSLQPEDFATYYCQQHYTTPPTFGQGTKVEIK      107
C1_VL_0-01        RFSGSRSGTDFTLTISSLQPEDFATYYCQQHYTTPPTFGQGTKVEIK      107
C2_VL_0-01        RFSGSRSGTDFTLTISSLQPEDFATYYCQQHYTTPPTFGQGTKVEIK      107
C3_VL_0-01        RFSGSRSGTDFTLTISSLQPEDFATYYCQQHYTTPPTFGQGTKVEIK      107
C4_VL_0-01        RFSGSRSGTDFTLTISSLQPEDFATYYCQQHYTTPPTFGQGTKVEIK      107
C5_VL_0-01        RFSGSRSGTDFTLTISSLQPEDFATYYCQQHYTTPPTFGQGTKVEIK      107
C6_VL_0-01        RFSGSRSGTDFTLTISSLQPEDFATYYCQQHYTTPPTFGQGTKVEIK      107
C7_VL_0-01        RFSGSRSGTDFTLTISSLQPEDFATYYCQQHYTTPPTFGQGTKVEIK      107
C8_VL_0-01        RFSGSRSGTDFTLTISSLQPEDFATYYCQQHYTTPPTFGQGTKVEIK      107
C9_VL_0-01        RFSGSRSGTDFTLTISSLQPEDFATYYCQQHYTTPPTFGQGTKVEIK      107
C10_VL_0-01       RFSGSRSGTDFTLTISSLQPEDFATYYCQQHYTTPPTFGQGTKVEIK      107
                  ***********************************************
```

Multiple alignment of 10 VH 0.0001% Sorted clones

```
CLUSTAL O(1.2.4) multiple sequence alignment


Herceptin_VH        EVQLVESGGGLVQPGGSLRLSCAASGFNIKDTYIHWVRQAPGKGLEWVARIYPTNGYTRY     60
C1_VH_0-0001        EVQLVESGGGLVQPGGSLRLSCAASGFNIKDTYIHWVRQAPGKGLEWVARIYPTNGYTRY     60
C2_VH_0-0001        EVQLVESGGGLVQPGGSLRLSCAASGFNIKDTYIHWVRQAPGKGLEWVARIYPTNGYTRY     60
C3_VH_0-0001        EVQLVESGGGLVQPGGSLRLSCAASGFNIKDTYIHWVRQAPGKGLEWVARIYPTNGYTRY     60
C4_VH_0-0001        EVQLVESGGGLVQPGGSLRLSCAASGFNIKDTYIHWVRQAPGKGLEWVARIYPTNGYTRY     60
C5_VH_0-0001        EVQLVESGGGLVQPGGSLRLSCAASGFNIKDTYIHWVRQAPGKGLEWVARIYPTNGYTRY     60
C6_VH_0-0001        EVQLVESGGGLVQPGGSLRLSCAASGFNIKDTYIHWVRQAPGKGLEWVARIYPTNGYTRY     60
C7_VH_0-0001        EVQLVESGGGLVQPGGSLRLSCAASGFNIKDTYIHWVRQAPGKGLEWVARIYPTNGYTRY     60
C8_VH_0-0001        EVQLVESGGGLVQPGGSLRLSCAASGFNIKDTYIHWVRQAPGKGLEWVARIYPTNGYTRY     60
C9_VH_0-0001        EVQLVESGGGLVQPGGSLRLSCAASGFNIKDTYIHWVRQAPGKGLEWVARIYPTNGYTRY     60
C10_VH_0-0001       EVQLVESGGGLVQPGGSLRLSCAASGFNIKDTYIHWVRQAPGKGLEWVARIYPTNGYTRY     60
                    ************************************************************

Herceptin_VH        ADSVKGRFTISADTSKNTAYLQMNSLRAEDTAVYYCSRWGGDGFYAMDYWGQGTLVTVSS     120
C1_VH_0-0001        ADSVKGRFTISADTSKNTAYLQMNSLRAEDTAVYYCSRWGGDGFYAMDYWGQGTLVTVSS     120
C2_VH_0-0001        ADSVKGRFTISADTSKNTAYLQMNSLRAEDTAVYYCSRWGGDGFYAMDYWGQGTLVTVSS     120
C3_VH_0-0001        ADSVKGRFTISADTSKNTAYLQMNSLRAEDTAVYYCSRWGGDGFYAMDYWGQGTLVTVSS     120
C4_VH_0-0001        ADSVKGRFTISADTSKNTAYLQMNSLRAEDTAVYYCSRWGGDGFYAMDYWGQGTLVTVSS     120
C5_VH_0-0001        ADSVKGRFTISADTSKNTAYLQMNSLRAEDTAVYYCSRWGGDGFYAMDYWGQGTLVTVSS     120
C6_VH_0-0001        ADSVKGRFTISADTSKNTAYLQMNSLRAEDTAVYYCSRWGGDGFYAMDYWGQGTLVTVSS     120
C7_VH_0-0001        ADSVKGRFTISADTSKNTAYLQMNSLRAEDTAVYYCSRWGGDGFYAMDYWGQGTLVTVSS     120
C8_VH_0-0001        ADSVKGRFTISADTSKNTAYLQMNSLRAEDTAVYYCSRWGGDGFYAMDYWGQGTLVTVSS     120
C9_VH_0-0001        ADSVKGRFTISADTSKNTAYLQMNSLRAEDTAVYYCSRWGGDGFYAMDYWGQGTLVTVSS     120
C10_VH_0-0001       ADSVKGRFTISADTSKNTAYLQMNSLRAEDTAVYYCSRWGGDGFYAMDYWGQGTLVTVSS     120
                    ************************************************************
```

Multiple alignment of 10 VL 0.0001% Sorted clones

```
CLUSTAL O(1.2.4) multiple sequence alignment


Herceptin           DIQMTQSPSSLSASVGDRVTITCRASQDVNTAVAWYQQKPGKAPKLLIYSASFLYSEVPS     60
C1_VL_0-0001        DIQMTQSPSSLSASVGDRVTITCRASQDVNTAVAWYQQKPGKAPKLLIYSASFLYSEVPS     60
C2_VL_0-0001        DIQMTQSPSSLSASVGDRVTITCRASQDVNTAVAWYQQKPGKAPKLLIYSASFLYSEVPS     60
C3_VL_0-0001        DIQMTQSPSSLSASVGDRVTITCRASQDVNTAVAWYQQKPGKAPKLLIYSASFLYSEVPS     60
C4_VL_0-0001        DIQMTQSPSSLSASVGDRVTITCRASQDVNTAVAWYQQKPGKAPKLLIYSASFLYSEVPS     60
C5_VL_0-0001        DIQMTQSPSSLSASVGDRVTITCRASQDVNTAVAWYQQKPGKAPKLLIYSASFLYSEVPS     60
C6_VL_0-0001        DIQMTQSPSSLSASVGDRVTITCRASQDVNTAVAWYQQKPGKAPKLLIYSASFLYSEVPS     60
C7_VL_0-0001        DIQMTQSPSSLSASVGDRVTITCRASQDVNTAVAWYQQKPGKAPKLLIYSASFLYSEVPS     60
C8_VL_0-0001        DIQMTQSPSSLSASVGDRVTITCRASQDVNTAVAWYQQKPGKAPKLLIYSASFLYSEVPS     60
C9_VL_0-0001        DIQMTQSPSSLSASVGDRVTITCRASQDVNTAVAWYQQKPGKAPKLLIYSASFLYSEVPS     60
C10_VL_0-0001       DIQMTQSPSSLSASVGDRVTITCRASQDVNTAVAWYQQKPGKAPKLLIYSASFLYSEVPS     60
                    ************************************************************

Herceptin           RFSGSRSGTDFTLTISSLQPEDFATYYCQQHYTTPPTFGQGTKVEIK   107
C1_VL_0-0001        RFSGSRSGTDFTLTISSLQPEDFATYYCQQHYTTPPTFGQGTKVEIK   107
C2_VL_0-0001        RFSGSRSGTDFTLTISSLQPEDFATYYCQQHYTTPPTFGQGTKVEIK   107
C3_VL_0-0001        RFSGSRSGTDFTLTISSLQPEDFATYYCQQHYTTPPTFGQGTKVEIK   107
C4_VL_0-0001        RFSGSRSGTDFTLTISSLQPEDFATYYCQQHYTTPPTFGQGTKVEIK   107
C5_VL_0-0001        RFSGSRSGTDFTLTISSLQPEDFATYYCQQHYTTPPTFGQGTKVEIK   107
C6_VL_0-0001        RFSGSRSGTDFTLTISSLQPEDFATYYCQQHYTTPPTFGQGTKVEIK   107
C7_VL_0-0001        RFSGSRSGTDFTLTISSLQPEDFATYYCQQHYTTPPTFGQGTKVEIK   107
C8_VL_0-0001        RFSGSRSGTDFTLTISSLQPEDFATYYCQQHYTTPPTFGQGTKVEIK   107
C9_VL_0-0001        RFSGSRSGTDFTLTISSLQPEDFATYYCQQHYTTPPTFGQGTKVEIK   107
C10_VL_0-0001       RFSGSRSGTDFTLTISSLQPEDFATYYCQQHYTTPPTFGQGTKVEIK   107
                    ***********************************************
```

Supplementary Table S1

**PCR Primer Pairs for HC**	
Adalimumab HC F1	5' - ATGGAACTGGGTCTCCGCTG - 3'
IRES R2	5' - CGGCTTCGGCCAGTAACGTTA - 3'
**PCR Primer Pairs for LC**	
Trastuzumab LC F	5' - ATGGACATGAGGGTCCCTGC - 3'
IRES R2	5' - CGGCTTCGGCCAGTAACGTTA - 3'
